# Supplementary material for: Study protocol designed to investigate tumour response to calcium electroporation in cancers affecting the skin: a non-randomised phase II clinical trial
Source: BMJ Open. 2021 Jun 16;11(6):e046779. doi: 10.1136/bmjopen-2020-046779 (PMC8211082; doi:10.1136/bmjopen-2020-046779)
Supplement: Author's manuscript [file bmjopen-2020-046779.draft_revisions.pdf]

BMJ Open is committed to open peer review. As part of this commitment we make the peer review history of every article we publish publicly available.

When an article is published we post the peer reviewers' comments and the authors' responses online. We also post the versions of the paper that were used during peer review. These are the versions that the peer review comments apply to.

The versions of the paper that follow are the versions that were submitted during the peer review process. They are not the versions of record or the final published versions. They should not be cited or distributed as the published version of this manuscript.

BMJ Open is an open access journal and the full, final, typeset and author-corrected version of record of the manuscript is available on our site with no access controls, subscription charges or pay-per-view fees (http://bmjopen.bmj.com).

If you have any questions on BMJ Open's open peer review process please email info.bmjopen@bmj.com

Study protocol designed to investigate tumour response to calcium electroporation in cancers affecting the skin

Journal:	*BMJ Open*
Manuscript ID	bmjopen-2020-046779
Article Type:	Protocol
Date Submitted by the Author:	10-Nov-2020
Complete List of Authors:	Vissing, Mille; Roskilde Sygehus, Department of Clinical Oncology and Palliative Care<br>Ploen, John; Vejle Hospital/Odense University Hospital, Department of Oncology<br>Pervan, Mascha; Clinic for Gynaecology and Obstetrics University Medical Centre Schleswig-Holstein<br>Vestergaard, Kitt; University College Absalon Campus Slagelse<br>Schnefeldt, Mazen; Vejle Hospital/Odense University Hospital<br>Frandsen, Stine; Roskilde Sygehus, Department of Clinical Oncology and Palliative Care<br>Rafaelsen, Soeren; Vejle Hospital/Odense University Hospital, Department of Radiology<br>Lindhardt, Christina; University College Absalon Campus Slagelse<br>Jensen, Lars Henrik; Department of Oncology, Lillebaelt Hospital, Vejle; Danish Colorectal Cancer Center South, Center of Clinical Excellence, Vejle Hospital, Institute of Regional Health Research, University of Southern Denmark<br>Rody, Achim; Clinic for Gynaecology and Obstetrics, University Medical Centre Schleswig-Holstein, Lübeck<br>Gehl, Julie; Roskilde Sygehus, Department of Clinical Oncology and Palliative Care
Keywords:	ONCOLOGY, Dermatological tumours < ONCOLOGY, Adult oncology < ONCOLOGY, Adult palliative care < PALLIATIVE CARE, Breast tumours < ONCOLOGY

SCHOLARONE™
Manuscripts

I, the Submitting Author has the right to grant and does grant on behalf of all authors of the Work (as defined in the below author licence), an exclusive licence and/or a non-exclusive licence for contributions from authors who are: i) UK Crown employees; ii) where BMJ has agreed a CC-BY licence shall apply, and/or iii) in accordance with the terms applicable for US Federal Government officers or employees acting as part of their official duties; on a worldwide, perpetual, irrevocable, royalty-free basis to BMJ Publishing Group Ltd ("BMJ") its licensees and where the relevant Journal is co-owned by BMJ to the co-owners of the Journal, to publish the Work in this journal and any other BMJ products and to exploit all rights, as set out in our licence.

The Submitting Author accepts and understands that any supply made under these terms is made by BMJ to the Submitting Author unless you are acting as an employee on behalf of your employer or a postgraduate student of an affiliated institution which is paying any applicable article publishing charge ("APC") for Open Access articles. Where the Submitting Author wishes to make the Work available on an Open Access basis (and intends to pay the relevant APC), the terms of reuse of such Open Access shall be governed by a Creative Commons licence – details of these licences and which Creative Commons licence will apply to this Work are set out in our licence referred to above.

Other than as permitted in any relevant BMJ Author's Self Archiving Policies, I confirm this Work has not been accepted for publication elsewhere, is not being considered for publication elsewhere and does not duplicate material already published. I confirm all authors consent to publication of this Work and authorise the granting of this licence.

For peer review only

Study protocol designed to investigate tumour response to calcium electroporation in cancers affecting the skin

Mille Vissing (1,2), John Ploen (3), Mascha Pervan (4), Kitt Vestergaard (5), Mazen Schnefeldt (6), Stine K Frandsen (1), Soeren Rafael Rafaelsen (6), Christina Louise Lindhardt (5,7), Lars Henrik Jensen (3), Achim Rody (4), Julie Gehl* (1,2)

1. Center for Experimental Drug and Gene Electrotransfer (C*EDGE), Department of Clinical Oncology and Palliative Care, Zealand University Hospital, Roskilde and Næstved, Sygehusvej 10, 4000 Roskilde, Denmark
2. Department of Clinical Medicine, Faculty of Health and Medical Sciences, University of Copenhagen, Blegdamsvej 3B, 2200 Copenhagen, Denmark
3. Department of Oncology, University Hospital of Southern Denmark, Vejle, Beriderbakken 4, 7100 Vejle, Denmark
4. Department of Obstetrics and Gynecology, University Hospital Schleswig-Holstein, Campus Lübeck, Ratzeburger Allee 160, 23562 Lübeck, Germany
5. Absalon University College, Sorø, Slagelsevej 70, 4180 Sorø, Denmark
6. Department of Radiology, University Hospital of Southern Denmark, Vejle, Beriderbakken 4, 7100 Vejle, Denmark
7. Department of Geriatric Medicine, University of Southern Denmark, Odense, Kløvervænget 10, 5000 Odense C, Denmark

*Corresponding author: Prof. Julie Gehl, Center for Experimental Drug and Gene Electrotransfer (C*EDGE). Department of Clinical Oncology and Palliative Care, Zealand University Hospital, Sygehusvej 10, 4000 Roskilde, Denmark. E-mail: kgeh@regionsjaelland.dk.

For peer review only - http://bmjopen.bmj.com/site/about/guidelines.xhtml

For peer review only

Abstract

Introduction

Skin malignancy is a distressing problem for many patients with cancer and clinical management is often challenging. This article describes the protocol for the CaEP-R study designed to investigate tumour response to calcium electroporation and functions as a descriptive guide to calcium electroporation treatment of malignant tumours in the skin. Calcium electroporation is a local treatment which induces supra-physiological intracellular calcium levels by intra-tumoural calcium administration and application of electrical pulses. The pulses create transient membrane pores that allow diffusion of non-permeant calcium ions into target cells. High calcium levels may kill cancer cells whilst normal cells can restore homeostasis. Prior trials have found calcium electroporation to be safe and efficient in smaller cohorts of patients. This trial aims to include a larger multi-regional cohort of patients with different cancer diagnoses, and also to investigate treatment areas using magnetic resonance imaging (MRI) as well as assess impact on quality of life.

Methods and analysis

This non-randomized phase II multi-centre study aims to investigate response to calcium electroporation in 30 patients with cutaneous or subcutaneous malignancy, with planned enrolment of 10 patients at each of three centres. A primary endpoint of response after two months was decided upon based on short-term response rates observed in the first small clinical study. Secondary endpoints include visualising response to treatment using MRI. Furthermore quality of life will be investigated using questionnaires and qualitative interviews. Patient and public involvement

In this study patients and the public were not involved in the writing of the protocol. It is planned to remedy this in part by forming a panel of patients, from both patients with cancer that were part of the study, and patients that were not part of the study. It is the plan to engage this panel in the discussion of the outcomes of both the response to therapy, and the quality of life analysis, as well as in the final publication(s) from the study.

For peer review only - http://bmjopen.bmj.com/site/about/guidelines.xhtml

Ethics and dissemination

In this study, all patients will receive active treatment with calcium electroporation. All patients may continue systemic treatment during the study side effects are expected to be limited. Data will be published in peer reviewed journal. Data will also be made available to the public.

Trial registration

Trial registration: ClinicalTrials.gov, NCT04225767. Registered 13 January 2020,

EudraCT no: 2019-11-05.

CaEP-R is part of Changing Cancer Care supported by the European Fund for Regional Development.

Strengths and limitations of this study

*This study investigating calcium electroporation is a novel treatment for cutaneous tumours

*This study details procedures for calcium electroporation and tests the technology in a multicentre setting

*By including patients with tumours of any cancer histology information may be gained for different types of cancer

*This study is limited in size, 30 patients

*Standardizing treatment methods as described in this protocol which is deemed important for outcome

Keywords

Calcium, electroporation, skin, cancer, metastases, tumour response, MRI, qualitative interviews.

Background

Clinical challenges of cancer affecting the skin

Skin manifestation of malignancy is a distressing problem for a significant number of patients with cancer. The area of disease can vary in size from a few millimetres to extensive areas of the body, and surgical or

For peer review only - http://bmjopen.bmj.com/site/about/guidelines.xhtml

oncological management is often challenging. The tumours may develop during the last few months of life or be present for a number of years and often require palliative management [1] Calcium electroporation (CaEP) is a new, promising local treatment for cutaneous and subcutaneous malignancy. [2 3]

As CaEP can be easily implemented, the encouraging results of the first clinical trials [3 4] should be investigated in a larger cohort before being translated to a standardised clinical setting. This is the main investigative purpose of this study.

The following describes anti-cancer properties of calcium overloading using the electroporation technique and outlines preclinical discoveries as well as the first clinical experiences with calcium electroporation for types of solid tumours, before describing protocol design.

Electroporation can be used to induce calcium overload

A method that can locally increase the intracellular concentration of otherwise non-permeant molecules is electroporation. Application of short high-voltage pulses can create transient pores in cell membranes allowing molecules to diffuse in and out of targeted cells. The electroporation method can be used to increase the uptake of anti-neoplastic agents [5-8] and nucleotides e.g. DNA/RNA [9-11] as well as non-permeant electrolytes such as calcium.[12-14]

Effects on normal and malignant cells - Preclinical studies

Calcium is a non-permeant ion involved as a signalling electrolyte in various cellular processes such as gene transcription, proliferation, metabolism and apoptosis. Human eukaryote cells can restore calcium homeostasis by storing intracellular Ca^{2+} in cellular compartments, chelating Ca^{2+} to intracellular proteins and extruding Ca^{2+} from the cytosol (Figure 1).

Tumourigenesis often alters cell calcium homeostasis through up- and down-regulation of calcium pumps and –channels, calcium storage compartments as well as changes in the cytoskeleton and membrane repair.[15] As a result, induction of cell death in cancer cells may be observed following calcium overload (Figure 1).[16-19] Mitochondrial collapse, ATP depletion, and ultimately promotion of necrotic cell death, have been

For peer review only - http://bmjopen.bmj.com/site/about/guidelines.xhtml

observed in cancer cells following CaEP *in vitro* and *in vivo.* [12 13 20] A preclinical cell study demonstrated that calcium alone does not have any influence on cells in terms of decrease of ATP and cell death.[12 13] Preclinical studies have shown that CaEP is efficient in inducing cell death *in vitro* - and *in vivo* across several cancer histologies including human breast-, bladder-, lung- and colon cancer cell lines [12-14 21 22]. Normal cells and tissues have been observed to be significantly more resistant to treatment than cancer cells and tissues in structured models.[14 22 23]

Calcium electroporation follows the principles of electrochemotherapy

Electrochemotherapy (ECT) is a standard therapy for cutaneous metastases where electroporation is used locally to increase cellular uptake of bleomycin, an anti-neoplastic drug.[24 25] Electrochemotherapy is well tested in treatment of different malignancies with both systemic- and local administration of bleomycin. Bleomycin induces apoptosis in cancer cells when delivered by electroporation and although the mechanisms through which calcium overload induces cell death differs from that of bleomycin (e.g. necrosis), the clinical setup and pulse parameters used for ECT are applicable.[2] CaEP is a local treatment where calcium is administrated intra-tumourally after which electrical pulses are applied to the target area. Like ECT, calcium electroporation may be used for treatment of primary or secondary skin tumours or ulcerating malignant wounds such as basal- and squamous cell skin cancers, melanoma skin cancer, Kaposi's sarcoma, breast cancer skin metastasis or head-and-neck cancer recurrence.[4 8 26-28] The procedure is performed in either general or local anaesthesia depending on tumour location and size, as well as patient preference and institutional standard operating procedure.[24 29]

Calcium electroporation for tumours affecting the skin and mucosa – Clinical studies

The first small clinical trial using CaEP in a double-blinded randomized study investigated whether CaEP was non-inferior to ECT and found CaEP to be a safe and efficient treatment which was non-inferior to ECT. The trial included seven patients of which six patients had breast cancer and one patient had malignant melanoma. A total of 47 cutaneous metastases were treated of which 37 were randomized and evaluated for

For peer review only - http://bmjopen.bmj.com/site/about/guidelines.xhtml

response and 10 were used for biopsy. A complete response (CR) was obtained in 66% (12/18) of calcium electroporated metastases versus 68% (13/19) of metastases treated with ECT.[4]

A second study with similar pre-set criteria, included seven patients with cutaneous metastasis (six patients with malignant melanoma and one patient with breast cancer). The study supported the safety and effectiveness of CaEP. While a difference in rates of CR was observed between metastases treated with CaEP (22%) and ECT (40%), both rates were lower compared to the first small initial trial.[3 4]

Finally, CaEP was proven safe and efficient in a trial investigating CaEP for recurrent head and neck cancer (6 patients) with a local response rate in treated lesions of 50% (3 of 6) which is comparable to those of ECT. The treatment had limited side effects and one of the treated patients exhibited CR.[4 28]

Purpose of this study

The purpose of this study is to determine response to calcium electroporation in a large cohort of patients with cutaneous tumours of any histology. This study will also encompass response evaluation by MRI and quality of life analyses.

Methods and study design

Aim

The aim of this study is to investigate calcium electroporation as an anti-cancer treatment for patients with malignant tumours situated in the skin.

Design and setting

This is a non-randomized phase II trial aiming to include 30 patients with cutaneous malignancy of any type. The primary endpoint is to evaluate the clinical response rate of calcium electroporation treatment of malignant tumours of the skin at two months after treatment using clinical examination documented by clinical photography. A maximum of seven tumours up to 3 cm in largest diameter will be treated and

For peer review only - http://bmjopen.bmj.com/site/about/guidelines.xhtml

followed pr. patient. All patients will receive treatment in this non-randomized phase II study. If a patient requests treatment of more tumours or retreatment, this may be accommodated but data will not be registered beyond seven tumours pr. patient. Calcium electroporation will not be compared to other means of treatment. This study will investigate the response rate of calcium electroporation treatment of skin metastases and malignant wounds in a real-world setting, at three cancer centres in Northern Europe. Quality of life will be investigated in patients treated at Zealand University Hospital using qualitative interviews before, two and 12 months after treatment. At Vejle Hospital, MRI will be performed before and after treatment on day of treatment and again after two months.

Participants

We seek to include 30 patients (10 at each site) with cutaneous malignancy of any cancer type. Inclusion criteria in the trial are: 1) age older than 18 years of age; 2) ability to understand the participant information; 3) histologically verified cutaneous or subcutaneous cancer of any type and histology; 4) must have been offered other relevant standard treatment for their cancer disease; 5) progressive or stable disease is present after a medical treatment period of two months or more (endocrine therapy, chemotherapy, immunotherapy etc.); 6) current radiation therapy does not involve the treated area; 7) performance status ECOG/WHO ≤2; 8) at least one cutaneous or subcutaneous tumour measuring up to 3 cm; 9) if sexually active, use of safe contraception (contraceptive coil, deposit injection of gestagen, subdermal implantation, hormonal vaginal ring or transdermal patch); 10) signed informed consent.

Exclusion criteria are pregnancy or lactation. Trial subjects are withdrawn from the study if; the patient withdraws his or her consent; the disease progresses so that the patient is in need of another treatment; investigator deems that withdrawing is in the best interest of the patient.

Study Process

For peer review only - http://bmjopen.bmj.com/site/about/guidelines.xhtml

Preoperative assessment

After consent and inclusion in the study, data concerning medical history including cancer diagnosis, TNM classification, past- and ongoing treatments, demographic data, comorbidity, radiological data, pathology, clinical photos, electrocardiograms, blood tests (including haemoglobin, blood cell count, serum-calcium and C-reactive protein) and list of prescription medicine, will be recorded. Relevant data will be stored on the case report form and EasyTrial clinical trial management system (EasyTrial ApS, Aalborg, Denmark).

Photography

The treated areas will be photographed prior to treatment on day 0 and at follow up at 1, 2, 3, 4, 6 and 12 months after treatment. At each visit, an overview photo of the treatment sites will be taken, as well as photos of each target tumour (Figure 2).

Quality of Life assessment

Patient quality of life (QoL) is planned for evaluation before treatment, after two months and after one year, through using the EORTC (European Organisation for Research and Treatment of Cancer) certified QLQ-C15-PAL Core Questionnaire as well as carrying out qualitative interviews (in a subset of patients). The QLQ-C15-PAL Core questionnaires evaluate cancer-related symptoms on a scale from 1-4 (“not at all”- “very much”) as well as overall QoL on a scale from 1-7 (“very poor”-“excellent”).

A subset of patients treated at Zealand University Hospital will participate in a qualitative interview at month 0 (before treatment), 2 and 12, in order to assess treatment impact on the QoL of cancer patients treated with CaEP. The qualitative interview seeks to explore patients’ physical and emotional well-being before and after treatment as well as expectations and experience in relation to calcium electroporation treatment. The interviews will be conducted as semi-structured interviews face-to-face and as phone calls, based on interview guides.[30] The recorded interviews will be transcribed, and analysed in order to enlighten physical and emotional well-being emphasized as an everyday life phenomena to describe quality of life, as it is experienced and attributed meaning by the patients.

For peer review only - http://bmjopen.bmj.com/site/about/guidelines.xhtml

For peer review only

Anaesthesia

Paracetamol 1 g and/or lidocaine cream may be prescribed 1 hour preoperatively as a prophylactic analgesic for mild pain. Calcium electroporation will be performed using local anaesthesia with injection of lidocaine-epinephrine 1% peri-tumourally, although other anaesthesia may be used. A sufficient amount of local anaesthetics will be applied, in order to reach full anaesthesia of the target area, which is comparative to other small local surgical procedures (Figure 3).

The maximum recommended dose of local anaesthesia will not be exceeded (30 ml for lidocaine-epinephrine 1%). The procedure may be performed in general anaesthesia and muscular relaxant administered, according to common procedure of the institute or according to patient request. The equipment used in the CaEP procedure is described in Figure 4.

Calcium dose and tumour volume

Calcium is administered intra-tumourally in this experiment. Participants will be given a maximum of 20 ml calcium solution of 220 mmol/L administered intra-tumourally, equalling a total calcium dose of 4.4 mmol. The calcium dose in this trial will be analogous to the first phase II trials testing calcium electroporation on small cutaneous tumours.[3 4] Ten ml calcium chloride 500 mM is suspended in 12.7 ml isotonic NaCl solution to a calcium dose of 220 mmol/l. Mixing is performed bedside and countersigned by observer. The dose to tumour volume ratio is an adapted calculation from the ESOPE guidelines (European Standard Operating Procedure of Electrochemotherapy).[29 31] 1) Tumour with a diameter <0.5 cm: 1 ml/cm^3 tumour tissue; 2) Tumour with a diameter from 0.5 cm to 3 cm: 0.5 ml/cm^3 of tumour tissue.

Tumour volume will be measured, using a Vernier calliper, and calculated according to ESOPE by the following formula: $V = ab^2 \pi / 6$ (a = the longest tumour diameter in cm; b = the longest diameter perpendicular to a).

It is important that the entire tumour volume and surrounding tissue is treated, thus we chose to define a treatment margin of 3 mm around the tumour when injecting calcium in the electroporation treatment area

For peer review only - http://bmjopen.bmj.com/site/about/guidelines.xhtml

(Figure 3). Calcium will be administered intra-tumourally by manual injection with linear application upon needle retirement spaced around 5 mm, to ensure an equal distribution of calcium in the tumour (Figure 5). As the volume of peripheral tissue is infinitely variable (Figure 6 a-c), adequate volume of injected calcium chloride should be individually assessed by the treating clinician.

For practical purposes, a calcium solution (220 mmol/L) dose corresponding to half of tumour volume (ml) is distributed in the margin area. Due to differences in tumour situation in the cutaneous and subcutaneous layers, standardised dose of the tumour margin area can be a challenge (Figure 6 a-c). The intra-tumourally-injected volume and total injected dose for each tumour is noted.

Assuming a standard whole body extracellular volume (ECV) of 15 L (dependent on patient size), and a total calcium concentration of 2.2-2.55 mmol/L of which approximately half (1.18-1.32mmol/L) is unbound and metabolically active, a normal extracellular store of calcium is ~33-35 mmol of which 18-20 mmol is unbound [32]. Administering a maximum dose of 4.4 mmol calcium under the circumstances described above would lead to an increase in total calcium concentration from 2.55 mmol/L to 2.66 mmol/L. Although total calcium concentrations would rise, the concentration of free unbound calcium is tightly regulated by a buffer system primarily controlled by albumin, ensuring homeostasis [32]. Free extracellular calcium concentrations can under normal circumstances rise to 1.4 mmol/L with no apparent symptoms. Symptoms of hypercalcemia may occur when free calcium exceeds 1.6 mmol/L and includes increased thirst, frequent urination and abdominal pain (CTCAE 4.0). As previously mentioned, we expect the injected calcium to act locally, with an insignificant systemic rise in unbound calcium as observed in a previous study 6 hrs post CaEP for recurrent head and neck cancer, injecting up to 13 ml 225 mmol/ml $CaCl_2$ i.t.[28] The method is deemed safe with local injection of up to 360 mg $CaCl_2$ (or 40 ml $CaCl_2$ 220 mmol/L solution). Moreover, electroporation causes a local hypo-perfusion through vasoconstriction thus the injected calcium will linger in the electroporated area, trapped intracellularly as membranes reseal (Figure 1).[23]

For peer review only - http://bmjopen.bmj.com/site/about/guidelines.xhtml

Applied electric pulses

As we inject calcium locally to the target tumour and margin area, pulses will be administered immediately after in an adjacent fashion to ensure an even distribution of treatment throughout the tumour (Figure 5). The distributed electric field of a linear array needle electrode is illustrated in Figure 7.

We use the CE-certified Cliniporator (IGEA, Carpi, Italy) square wave pulse generator for electroporation, which delivers a series of eight consecutive pulses of 0.1 msec each with an amplitude of 1 kV/cm and a frequency of 1 Hz. Linear array electrodes (as used in this study) lead to a higher response rate in small cutaneous tumours.[33]

The maximum current delivered during treatment as measured by the pulse generator will be noted for each tumour.

MRI

On a subset of patients MRI may be used to assess response before (from 30 min up to 24 hours) and immediately after treatment (within 30 min up to 4 hrs), as well as after 2 months using diffusion-weighted magnetic resonance imaging (DW-MRI) as a method to monitor electroporated tissue, using the apparent diffusion coefficient (ADC). ADC is a measure of the magnitude of diffusion of water molecules within tissue, clinically calculated using DW-MRI and expressed in units of mm^2/s.[34-36]

Side effects

The side effects of calcium electroporation for cutaneous and subcutaneous metastases less than 3 cm in diameter have been described in previous studies.[3 4] When the electrical pulse is given, there is a short-term contraction of the underlying muscles. Patients treated under local anaesthesia can experience the muscle contraction as mildly unpleasant. Patients under general anaesthesia will not register this contraction. The electric pulses last less than a second, and muscle contractions last for only the period in which the electric pulses are given. Experiences from treating small tumours with electrochemotherapy and calcium electroporation show that the treated area can initially become erythematous and swollen, but symptoms

For peer review only - http://bmjopen.bmj.com/site/about/guidelines.xhtml

usually fade within hours to days. The area can subsequently become necrotic but generally heals within 6-10 weeks. In a few cases, infection may occur in the treatment area. In case of infection, the patient may require antibiotics according to local guidelines.[3 4]

The treated area will usually require a bandage the first few days postoperatively because of slight oozing. Any postoperative pain may be treated according to local guidelines e.g. 1 g paracetamol p.n. A doctor will control the treated area at follow-up.

Adverse Events (AE) and Serious Adverse Events (SAE) will be evaluated and graded according to CTCAE version 4.0. In view of the severity of metastatic cancer disease, there are certain conditions that are defined as SAEs but are not reported as such in this study, e.g. voluntary hospitalization and surgery as treatment of the underlying cancer. Patients are encouraged to take any prescribed medications before, during and after treatment. The results of the study will in this instance be more translational to a real world setting.

Any anticoagulant medication may also be continued, as the minor blood spill in treatment of tumours up to 3 cm in diameter is expected to be manageable.

Evaluation of treatment response

Response will be evaluated according to the modified RECIST guideline [37] and defined as: Complete Response (CR) – disappearance of the lesion; Partial Response (PR) – at least 30% decrease in the largest diameter of the lesion; Progressive Disease (PD) – at least 20% increase in the largest diameter of the lesion; Stable Disease (SD) – neither 30% decrease nor 20% increase of the largest diameter of the lesion.

The primary endpoint is to assess response rate after 2 months. The two month time point was based on response rates observed in the first clinical trial by Falk et al.[4] Response rate will be defined as number of responding lesions (partial or complete response) relative to treated lesions evaluated by changes in size (mm) by clinical examination with calliper measurement. Tumour response will be further documented using clinical photography.

Response will be assessed by clinical examination at 1, 2, 3, 4, 6 and 12 months of follow-up after treatment. Changes in tumour size will be registered for each target lesion and documented by digital photography. If

For peer review only - http://bmjopen.bmj.com/site/about/guidelines.xhtml

treated areas present a crusted wound, the crust and any residual tumour is measured and clinical responsiveness noted.

To evaluate long-term response, punch biopsies (preferably 4 mm) will be taken from the centre and edge of the treated areas one year after treatment. Biopsies of the tumour area will be stored as formalin-fixated, paraffin-embedded tissue blocks. Analyses will assess tumour- and surrounding tissue response to treatment histological analyses (e.g. percent tumour cells in sample and fibrosis). Samples will not be taken before 12 months follow up to exclude response due to tissue loss from biopsies.

We aim to investigate any sign of systemic immunologic response from any routine scans (MRI, PET-CT etc.) before and after treatment in the inclusion period by monitoring tumour size and TNM stage. Response rates and response duration according to tumour histology will be compared as well as the rate of response for each individual patient.

For peer review only

Statistical power calculation and definition of cohort

This is a phase II non-controlled study. The primary endpoint is to evaluate tumour response (overall response which includes partial- and complete responses) two months after CaEP treatment evaluated by clinical examination and documented by photography. The appropriate sample size is calculated to determine the minimum number of subjects that need to be enrolled in this study, in order to have sufficient statistical power in detecting calcium electroporation treatment effect.

Based on the response rate from the initial but small study with calcium electroporation treatment we predicted a mean complete response rate of 66%.[4] In order to define our cohort, a response rate of no treatment is estimated at 0%. To detect a 66% difference with a power of 90%, eight patients should be included. As we aim to support earlier studies in a real-world setting, 10 patients from each involved cancer centre will be included. Because our primary end point is follow-up after 2 months we cautiously predict that 2 patients out of 10 will be excluded or omitted from the study. We thereby expect 24 evaluable patients at primary end point with 95% CI [51.64, 80.36] at an SD estimated at 36%. Off-study patients will not lead to further inclusion.

For peer review only - http://bmjopen.bmj.com/site/about/guidelines.xhtml

At least one tumour will be treated in each patient, however, up to 7 tumours per patient may be included in the study.

We aim to calculate the response rate of calcium electroporation across all treated tumours.

Discussion

Considerations regarding study design

In this study we seek to illuminate the treatment effect of CaEP in a larger cohort of patients with cutaneous or subcutaneous malignancy of different histological features. In the planned study, Vejle Hospital will use MRI to visualise and quantify the immediate effects of CaEP in a subgroup of patients. This method could provide the first imaging of immediate skin tumour response to treatment with CaEP and assess the treated area perioperatively.

Why all cancers?

As preclinical studies have shown response to treatment in all tested cancer cell-lines of different types [13 14 21-23], clinical studies using CaEP for any solid tumours are warranted. As the treatment is safe and has not led to disease progression or increased tumour growth compared to controls in any preclinical or clinical studies, we expect CaEP treatment to be safe in treatment of cutaneous or subcutaneous malignant tumours of any histology.

Multi-centre setting

As the treatment modality may be easily implemented, the encouraging results of the first clinical trials need to be supported in a larger cohort before being feasibly translated to a standard clinical setting. Conducting a Phase II trial in a multicentre setting, operating and including at three cancer centres, presents the possibility of further investigating this novel treatment modality in a larger cohort. One team has previous experience with CaEP, another with ECT and one centre has no previous experience with electroporation treatment.

For peer review only - http://bmjopen.bmj.com/site/about/guidelines.xhtml

Tumour response determination and MRI

We chose tumour response after two months based on responses observed in the study by Falk et al investigating calcium electroporation for cutaneous metastasis.[4] In an attempt to improve outcomes and minimise risk of recurrence, we include a 3 mm treatment margin around the tumour and exclusively use linear array needle electrodes, which are superior in treatment of smaller tumours in the skin, to plate and hexagonal needle electrodes.[33]

Along with response evaluation based on changes in tumour size, we will also have photographic documentation of the progression of treated cancer tumours in the skin following calcium electroporation. The results of MR may uncover whether cell swelling for CaEP can be visualized after treatment of malignant tumours of the skin, thus aiding postoperative verification of treatment area. Follow up MR scans may also help visualize differences in healing outcomes and mechanisms in subcutaneous and cutaneous tumours respectively.

Although different cancer entities will represent smaller cohorts, it will be interesting to compare response rates and response duration according to tumour type. When we analyse our results we further aim to evaluate the importance of previous irradiation by investigating response (overall, as well as complete and partial) depending whether the treated tumour was in a previously irradiated area.

Qualitative interviews

A subset of patients treated at Zealand University Hospital will participate in qualitative interviews, in the first attempt to provide more detailed and nuanced data regarding QoL in patients treated with CaEP than standardised questionnaires.[38] The data may provide important knowledge about CaEP treatment's impact on the QoL of patients with cancer tumours affecting the skin, as well as patient perspective on CaEP treatment. Qualitative data may further uncover concerns and priorities that distinguish cancer patients with skin malignancy from other patient populations and help to adequately capture patients' experience.

For peer review only - http://bmjopen.bmj.com/site/about/guidelines.xhtml

Limitations

The proposed study is a clinical prospective study investigating a heterogeneous group of patients with malignancies at different disease stages. The included patients will vary in number and type of previous and ongoing therapies which either directly or indirectly target the included tumours. The number of included patients is limited and no control group will be included. With expected inclusion of 30 patients, statistical conclusions will need to be drawn based on even smaller subgroups of tumour entities. Furthermore, the tumours to be included in this study will not have responded to standard therapies after two months and may represent a cohort of treatment-resistant cancers with lower response rates than in a real world setting.

The clinical significance of the effect of CaEP on QoL will be limited to patients with cutaneous metastases up to 3 cm in diameter. Stigmatising or distressing symptoms such as suppuration, malodour and pain, often correlate with more advanced skin tumours.

Perspectives

Our hypothesis is that CaEP is a safe and effective treatment for skin metastases and other malignant tumours of the skin with limited side effects. The treatment may result in a reduction of tumour volume in the treated areas and possibly improve QoL for cancer patients.

Calcium electroporation is a novel anti-cancer treatment and this study aims to verify response rates in a larger prospective cohort across three cancer centres planning inclusion of patients with different types of cutaneous or subcutaneous cancer disease.

The response rates of metastatic disease could be important for future studies investigating calcium electroporation as a treatment modality for malignancy of internal organs. Using calcium electroporation as pre- or post-surgical treatment or combined with interventional radiology could be interesting to explore in future studies. Using calcium in procedures using irreversible electroporation, an established technique used for tissue ablation, could potentially improve outcomes in cancer surgery as it has been shown that there is a perimeter of tissue that undergoes reversible electroporation around ablated treatment areas.[39]

For peer review only - http://bmjopen.bmj.com/site/about/guidelines.xhtml

If supported, calcium electroporation will be a readily accessible efficient treatment for appurtenant facilities equipped with an electroporator. Using calcium in electroporation treatments may benefit many clinicians and patients alike, as calcium is a safe, naturally occurring electrolyte with low cost, easy handling and limited side effects.

List of abbreviations

EP: Electroporation; CaEP: Calcium electroporation; CaEP-R: Study title; $CaCl_2$: Calcium chloride; QoL: Quality of life; ECT: Electrochemotherapy, ECV: Extracellular volume; ATP: Adenosine triphosphate; CI: Confidence interval; SD: Standard deviation; EORTC: European Organisation for Research and Treatment of Cancer; AE: Adverse event; SAE: Serious adverse event; PR: Partial response; CR: Complete response; PD: Progressive disease; SD: Stable disease.

Declarations

Ethics approval and consent to participate

The trial will be conducted in accordance with the official version of the Declaration of Helsinki and in agreement with the ICH directions for Good Clinical Practice and the respective rules and regulations in Denmark and Germany. The trial is approved by all relevant authorities in Denmark, and pending approval from relevant authorities in Germany. Accepted by the Danish Ethics Committee (Den Videnskabsetiske Komite for Region Sjælland), December 13, 2019 (case no: SJ-810). EudraCT no. 2019-11-05, Data Protection Agency no. REG-115-2019, ClinicalTrials.gov Identifier: NCT04225767. Participation in the study requires signed informed consent.

For peer review only - http://bmjopen.bmj.com/site/about/guidelines.xhtml

Competing interests

JG and SKF are co-inventors of a patent regarding calcium electroporation.

Funding

The Interreg consortium Changing Cancer Care (CCC) 094-1.1-18 funded by the European Regional Development Fund has supported the project.

Authors' contributions

MV was the primary author of the protocol and the manuscript. The study concept was initially designed by MV and JG, expanded for MR, QoL and execution in Germany by LHJ, SRR, CLL, MP and AR. MV, SKF, KV, MS and JG contributed to the manuscript. All authors read and approved the final manuscript.

Acknowledgements

Not applicable.

References

1. van Leeuwen BL, Houwerzijl M, Hoekstra HJ. Educational tips in the treatment of malignant ulcerating tumours of the skin. *Eur J Surg Oncol* 2000;26(5):506-8. doi: 10.1053/ejso.1999.0931
2. Falk H, Matthiessen LW, Wooler G, et al. Calcium electroporation for treatment of cutaneous metastases; a randomized double-blinded phase II study, comparing the effect of calcium electroporation with electrochemotherapy. *Acta Oncol* 2018;57(3):311-19. doi: 10.1080/0284186X.2017.1355109
3. Agoston D, Baltas E, Ocsai H, et al. Evaluation of Calcium Electroporation for the Treatment of Cutaneous Metastases: A Double Blinded Randomised Controlled Phase II Trial. *Cancers (Basel)* 2020;12(1) doi: 10.3390/cancers12010179
4. Falk H, Matthiessen LW, Wooler G, et al. Calcium electroporation for treatment of cutaneous metastases; a randomized double-blinded phase II study, comparing the effect of calcium electroporation with electrochemotherapy. *Acta Oncol* 2017:1-9. doi: 10.1080/0284186X.2017.1355109
5. Mir LM, Banoun H, Paoletti C. Introduction of definite amounts of nonpermeant molecules into living cells after electropermeabilization: direct access to the cytosol. *Experimental Cell Research* 1988;175(1):15-25.

For peer review only - http://bmjopen.bmj.com/site/about/guidelines.xhtml

For peer review only

6. Sersa G, Cemazar M, Miklavcic D. Antitumor effectiveness of electrochemotherapy with cis-diamminedichloroplatinum(II) in mice. *Cancer Research* 1995;55(15):3450-55.
7. Gothelf A, Mir LM, Gehl J. Electrochemotherapy: results of cancer treatment using enhanced delivery of bleomycin by electroporation. *Cancer Treatment Reviews* 2003;29(5):371-87.
8. Clover AJP, Salwa SP, Bourke MG, et al. Electrochemotherapy for the treatment of primary basal cell carcinoma; A randomised control trial comparing electrochemotherapy and surgery with five year follow up. *Eur J Surg Oncol* 2020;46(5):847-54. doi: 10.1016/j.ejso.2019.11.509
9. Neumann E, Schaefer-Ridder M, Wang Y, et al. Gene transfer into mouse lyoma cells by electroporation in high electric fields. *EMBO J* 1982;1(7):841-45.
10. Heller L, Pottinger C, Jaroszeski MJ, et al. In vivo electroporation of plasmids encoding GM-CSF or interleukin-2 into existing B16 melanomas combined with electrochemotherapy induces long-term antitumour immunity. *Melanoma Res* 2000;10(0960-8931 SB - IM):577-83.
11. Sersa G, Teissie J, Cemazar M, et al. Electrochemotherapy of tumors as in situ vaccination boosted by immunogene electrotransfer. *Cancer Immunol Immunother* 2015;64(10):1315-27. doi: 10.1007/s00262-015-1724-2
12. Frandsen SK, Gissel H, Hojman P, et al. Direct therapeutic applications of calcium electroporation to effectively induce tumor necrosis. *Cancer Res* 2012;72(6):1336-41. doi: 10.1158/0008-5472.CAN-11-3782
13. Hansen EL, Sozer EB, Romeo S, et al. Dose-dependent ATP depletion and cancer cell death following calcium electroporation, relative effect of calcium concentration and electric field strength. *PLoS One* 2015;10(4):e0122973. doi: 10.1371/journal.pone.0122973
14. Frandsen SK, Kruger MB, Mangalanathan UM, et al. Normal and Malignant Cells Exhibit Differential Responses to Calcium Electroporation. *Cancer Res* 2017;77(16):4389-401. doi: 10.1158/0008-5472.CAN-16-1611
15. Frandsen SK, Vissing M, Gehl J. A Comprehensive Review of Calcium Electroporation -A Novel Cancer Treatment Modality. *Cancers (Basel)* 2020;12(2) doi: 10.3390/cancers12020290
16. Aung CS, Kruger WA, Poronnik P, et al. Plasma membrane Ca2+-ATPase expression during colon cancer cell line differentiation. *Biochem Biophys Res Commun* 2007;355(4):932-6. doi: 10.1016/j.bbrc.2007.02.050
17. Papp B, Brouland JP, Arbabian A, et al. Endoplasmic reticulum calcium pumps and cancer cell differentiation. *Biomolecules* 2012;2(1):165-86. doi: 10.3390/biom2010165
18. Rizzuto R, De Stefani D, Raffaello A, et al. Mitochondria as sensors and regulators of calcium signalling. *Nat Rev Mol Cell Biol* 2012;13(9):566-78. doi: 10.1038/nrm3412
19. Bonora M, Bononi A, De Marchi E, et al. Role of the c subunit of the FO ATP synthase in mitochondrial permeability transition. *Cell Cycle* 2013;12(4):674-83. doi: 10.4161/cc.23599
20. Gibot L, Montigny A, Baaziz H, et al. Calcium Delivery by Electroporation Induces In Vitro Cell Death through Mitochondrial Dysfunction without DNA Damages. *Cancers (Basel)* 2020;12(2) doi: 10.3390/cancers12020425
21. Frandsen SK, Gissel H, Hojman P, et al. Calcium electroporation in three cell lines: a comparison of bleomycin and calcium, calcium compounds, and pulsing conditions. *Biochim Biophys Acta* 2014;1840(3):1204-8. doi: 10.1016/j.bbagen.2013.12.003
22. Frandsen SK, Gibot L, Madi M, et al. Calcium Electroporation: Evidence for Differential Effects in Normal and Malignant Cell Lines, Evaluated in a 3D Spheroid Model. *PLoS One* 2015;10(12):e0144028. doi: 10.1371/journal.pone.0144028
23. Staresinic B, Jesenko T, Kamensek U, et al. Effect of calcium electroporation on tumour vasculature. *Sci Rep* 2018;8(1):9412. doi: 10.1038/s41598-018-27728-z
24. Gehl J, Sersa G, Garbay J, et al. Results of the ESOPE (European Standard Operating Procedures on Electrochemotherapy) study: Efficient, highly tolerable and simple palliative treatment of cutaneous and subcutaneous metastases from cancers of any histology. *JClinOncol* 2006;24(188)

For peer review only - http://bmjopen.bmj.com/site/about/guidelines.xhtml

25. Cadossi R, Ronchetti M, Cadossi M. Locally enhanced chemotherapy by electroporation: clinical experiences and perspective of use of electrochemotherapy. *Future Oncol* 2014;10(5):877-90. doi: 10.2217/fon.13.235
26. Falk H, Lambaa S, Johannesen HH, et al. Electrochemotherapy and calcium electroporation inducing a systemic immune response with local and distant remission of tumors in a patient with malignant melanoma - a case report. *Acta Oncol* 2017;56(8):1126-31. doi: 10.1080/0284186X.2017.1290274
27. Szewczyk A, Gehl J, Daczewska M, et al. Calcium electroporation for treatment of sarcoma in preclinical studies. *Oncotarget* 2018;9(14):11604-18. doi: 10.18632/oncotarget.24352
28. Plaschke CC, Gehl J, Johannesen HH, et al. Calcium electroporation for recurrent head and neck cancer: A clinical phase I study. *Laryngoscope Investig Otolaryngol* 2019;4(1):49-56. doi: 10.1002/lio2.233
29. Gehl J, Sersa G, Matthiessen LW, et al. Updated standard operating procedures for electrochemotherapy of cutaneous tumours and skin metastases. *Acta Oncol* 2018;57(7):874-82. doi: 10.1080/0284186X.2018.1454602
30. Glesne C. Becoming Qualitative Researchers. An Introduction. 5. ed: Pearson 2016.
31. Marty M, Sersa G, Garbay JR, et al. Electrochemotherapy - An easy, highly effective and safe treatment of cutaneous and subcutaneous metastases: Results of ESOPE (European Standard Operating Procedures of Electrochemotherapy) study. *Ejc Supplements* 2006;4(11):3-13.
32. Baird GS. Ionized calcium. *Clin Chim Acta* 2011;412(9-10):696-701. doi: 10.1016/j.cca.2011.01.004
33. Clover J, Terlizzi Fd, Bertino G, et al. Electrochemotherapy in the treatment of cutaneous malignancy; outcomes and subgroup analysis from the cumulative results from the pan- European InspECT Database for 2482 lesions in 987 patients (2008-2019). *European Journal of Cancer* 2020
34. Sener RN. Diffusion MRI: apparent diffusion coefficient (ADC) values in the normal brain and a classification of brain disorders based on ADC values. *Comput Med Imaging Graph* 2001;25(4):299-326. doi: 10.1016/s0895-6111(00)00083-5
35. Mahmood F, Hansen RH, Agerholm-Larsen B, et al. Diffusion-weighted MRI for verification of electroporation-based treatments. *J Membr Biol* 2011;240(3):131-8. doi: 10.1007/s00232-011-9351-0
36. Mahmood F, Hansen RH, Agerholm-Larsen B, et al. Detection of electroporation-induced membrane permeabilization states in the brain using diffusion-weighted MRI. *Acta Oncol* 2015;54(3):289-97. doi: 10.3109/0284186X.2014.991045
37. Eisenhauer EA, Therasse P, Bogaerts J, et al. New response evaluation criteria in solid tumours: revised RECIST guideline (version 1.1). *Eur J Cancer* 2009;45(2):228-47. doi: 10.1016/j.ejca.2008.10.026
38. Bhat TS, Herbosa CM, Rosenberg AR, et al. Current measures are not sufficient: an interview-based qualitative assessment of quality of life in cutaneous T-cell lymphoma. *Br J Dermatol* 2020 doi: 10.1111/bjd.19298
39. Hjouj M, Last D, Guez D, et al. MRI study on reversible and irreversible electroporation induced blood brain barrier disruption. *PLoS One* 2012;7(1932-6203 (Electronic)):e42817-e17.
40. Berridge MJ, Bootman MD, Roderick HL. Calcium signalling: dynamics, homeostasis and remodelling. *Nat Rev Mol Cell Biol* 2003;4(7):517-29. doi: 10.1038/nrm1155
41. Kania E, Pajak B, Orzechowski A. Calcium homeostasis and ER stress in control of autophagy in cancer cells. *Biomed Res Int* 2015;2015:352794. doi: 10.1155/2015/352794
42. Marchi S, Pinton P. Alterations of calcium homeostasis in cancer cells. *Curr Opin Pharmacol* 2016;29:1-6. doi: 10.1016/j.coph.2016.03.002
43. Frandsen SK, McNeil AK, Novak I, et al. Difference in Membrane Repair Capacity Between Cancer Cell Lines and a Normal Cell Line. *J Membr Biol* 2016;249(4):569-76. doi: 10.1007/s00232-016-9910-5
44. Calmels L, Al-Sakere B, Ruaud JP, et al. In vivo MRI follow-up of murine tumors treated by electrochemotherapy and other electroporation-based treatments. *Technol Cancer Res Treat* 2012;11(6):561-70.

For peer review only - http://bmjopen.bmj.com/site/about/guidelines.xhtml

45. Falk H, Forde PF, Bay ML, et al. Calcium electroporation induces tumor eradication, long-lasting immunity and cytokine responses in the CT26 colon cancer mouse model. *Oncoimmunology* 2017;6(5):e1301332. doi: 10.1080/2162402X.2017.1301332
46. Gehl J, Sorensen TH, Nielsen K, et al. In vivo electroporation of skeletal muscle: threshold, efficacy and relation to electric field distribution. *Biochim Biophys Acta* 1999;1428(2-3):233-40. doi: 10.1016/s0304-4165(99)00094-x

FIGURE 1

Schematic overview of known effects of calcium electroporation on normal cells and cancer cells

Electroporation pulse treatment creates transient pores in both cancer cells (bottom row) and normal cells (top row). In a high calcium concentration environment, the intracellular calcium level is greatly increased immediately after treatment.[13] After a few minutes, the plasma membrane reseals and both normal and malignant cells are overloaded with Ca^{2+}. Ca^{2+}-extrusion from the cytoplasm is carried out by Na^{+}-Ca^{2+} exchangers, plasma membrane Ca^{2+}-ATPase (PMCA) pumps and sarco-endoplasmic reticulum Ca^{2+}-ATPase (SERCA) pumps that transport Ca^{2+} into the extracellular space and into the endoplasmic reticulum (ER) lumen respectively, whilst consuming ATP in both normal and cancer cells [40]. Suppression of free cytosolic calcium is further facilitated by buffering in mitochondrial compartments and binding to Ca^{2+}-binding proteins. Calcium pumps and channels may be up- or downregulated in malignant cells, impairing calcium homeostasis.[16 17 41 42] Furthermore, cancer cells can have weakened membrane repair mechanisms.[43] Ca^{2+} overload induces mitochondrial dysfunction and critical ATP depletion in cancer cells.[13 20 43] These properties may act as mechanisms of calcium electroporation-induced cell death. As illustrated, electroporation may also cause cellular swelling.[44] Following calcium electroporation, normal cells have the ability to restore homeostasis while cancer cells are prone to necrotise. Cancer cell necrosis leads to release of a danger signal as well as cell remnants that may induce a local and/or systemic immune response.[45]

FIGURE 2

Pretreatment and follow up photography

For peer review only - http://bmjopen.bmj.com/site/about/guidelines.xhtml

Guide to overview photo of numbered target lesions (a) and photo of target lesion centrally in the image with longest diameter horizontally. Tumour marked proximally (violet) and adhesive ruler for scale 1 cm below the tumour at the bottom of the image (b). Tumours are numbered according to clinician preference, preferably with the most symptomatic tumour marked as tumour 1.

FIGURE 3

Treatment area

Local anaesthetic applied peri-tumourally in a rhomboid manner in an appropriate distance from the target area. For a total tumour treatment, the aim is to treat all the tumour with a 3 mm margin of clinically normal skin included in the target area. Blue-grey: anaesthetized area; Rose: tumour; Violet: Target margin.

FIGURE 4

Calcium electroporation equipment

Local anaesthetic is injected around the target area with 10 ml syringes (a). Calcium is injected to the target area with 1 ml syringes (tumours under 3 cm in largest diameter) (b). For larger tumours syringes 3-5 ml may be appropriate. Pulse treatment is applied using linear electrodes (c) connected to a pulse generator (d) with a foot paddle that allows arming the electrode and delivery of the electrical pulses (e). Pulse parameters and delivered pulses are shown on the generator screen (f).

FIGURE 5

Calcium electroporation procedure

Local anaesthetic is injected dermally around the tumour in a rhomboid manner with an appropriate distance from the target area (a). The calculated i.t. dose of calcium is injected into the tumour in a parallel fashion throughout the tumour. The margin area is then supplemented with calcium until the calcium is evenly distributed throughout the entire target area (b). The electrode is applied into the target area (c). Max treatment

For peer review only - http://bmjopen.bmj.com/site/about/guidelines.xhtml

For peer review only

depth is 3 cm and the needles should reach through the entire tumour depth and margin tissue below if possible. Electrode application (c) is repeated in an adjacent manner until the entire target area is treated (d). If multiple tumours are to be treated all targets are anaesthetized (a) first after which (b-d) is repeated for each target.

FIGURE 6

Cancer location in skin layers. Tumours may present as subcutaneous or cutaneous and involve some or all skin layers e.g. subcutaneous fat (a), dermis (b) and/or epidermis (c). If the epidermis is involved the tumours may present as ulcerating or fungating wounds.

Intact skin vs necrosis after CaEP. In most cases, subcutaneous tumours (a) will not ulcerate after calcium electroporation and skin will often appear intact after treatment. If treated tumours are situated as in (b) there may or may not be ulceration after treatment, depending on many factors such as patient healing potential and degree of invasion of the upper skin layers. Ulcerating or fungating tumours (c) may develop a necrotic, often crusted wound following treatment.

FIGURE 7

Distrubution of electric field

Linear array electrode for electroporation with 0.7 mm width 3 cm needles (IGEA) (a). Calculated E-field distribution at an applied voltage of 1.2 kV/cm for needle electrodes with two arrays of each four needles placed 4 mm apart (b). The spacing between the needles in the array is 2 mm. The small non-symmetric contour lines are due to limitations in the boundary conditions of the program, from Gehl et al.[46] Note that inoculation of living cancer cells during treatment may be avoided by irreversible electroporation (>1.2 kV/cm) and cell-kill zone of tissue adjacent to the electrodes (>1.6 kV/cm).

For peer review only - http://bmjopen.bmj.com/site/about/guidelines.xhtml

For peer review only

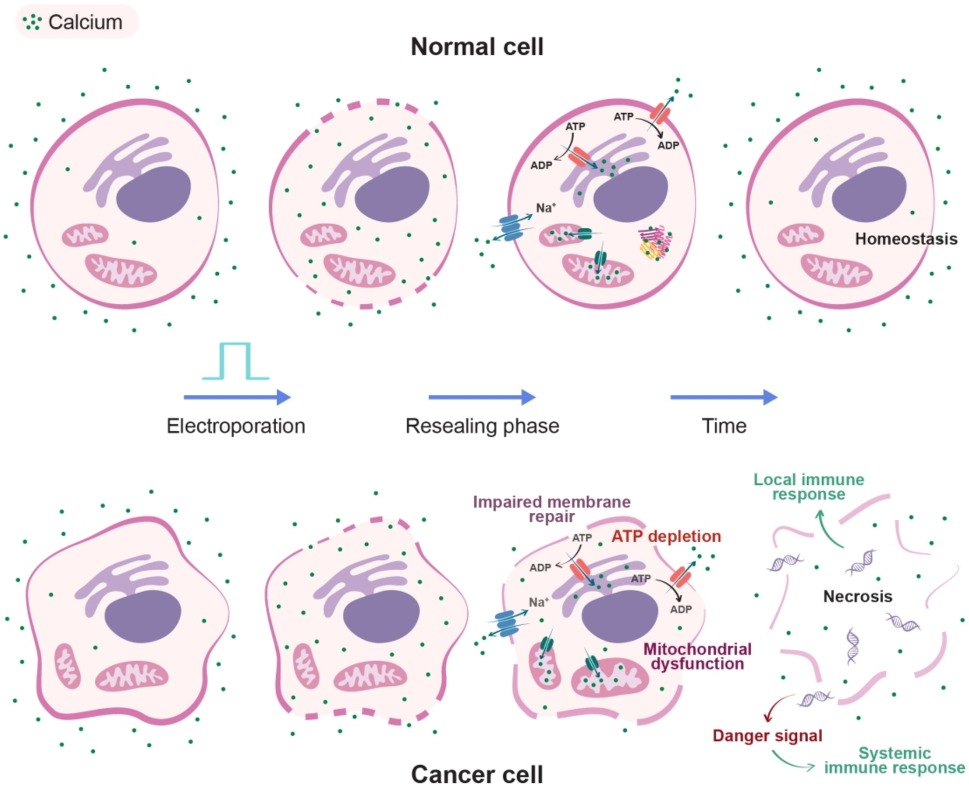

FIGURE 1

Schematic overview of known effects of calcium electroporation on normal cells and cancer cells

Electroporation pulse treatment creates transient pores in both cancer cells (bottom row) and normal cells (top row). In a high calcium concentration environment, the intracellular calcium level is greatly increased immediately after treatment.13 After a few minutes, the plasma membrane reseals and both normal and malignant cells are overloaded with Ca2+. Ca2+-extrusion from the cytoplasm is carried out by Na+-Ca2+ exchangers, plasma membrane Ca2+-ATPase (PMCA) pumps and sarco-endoplasmic reticulum Ca2+-ATPase (SERCA) pumps that transport Ca2+ into the extracellular space and into the endoplasmic reticulum (ER) lumen respectively, whilst consuming ATP in both normal and cancer cells 40. Suppression of free cytosolic calcium is further facilitated by buffering in mitochondrial compartments and binding to Ca2+-binding proteins. Calcium pumps and channels may be up- or downregulated in malignant cells, impairing calcium homeostasis.16 17 41 42 Furthermore, cancer cells can have weakened membrane repair mechanisms.43 Ca2+ overload induces mitochondrial dysfunction and critical ATP depletion in cancer cells.13 20 43 These properties may act as mechanisms of calcium electroporation-induced cell death. As illustrated, electroporation may also cause cellular swelling.44 Following calcium electroporation, normal cells have the ability to restore homeostasis while cancer cells are prone to necrotise. Cancer cell necrosis leads to release of a danger signal as well as cell remnants that may induce a local and/or systemic immune response.45

170x141mm (300 x 300 DPI)

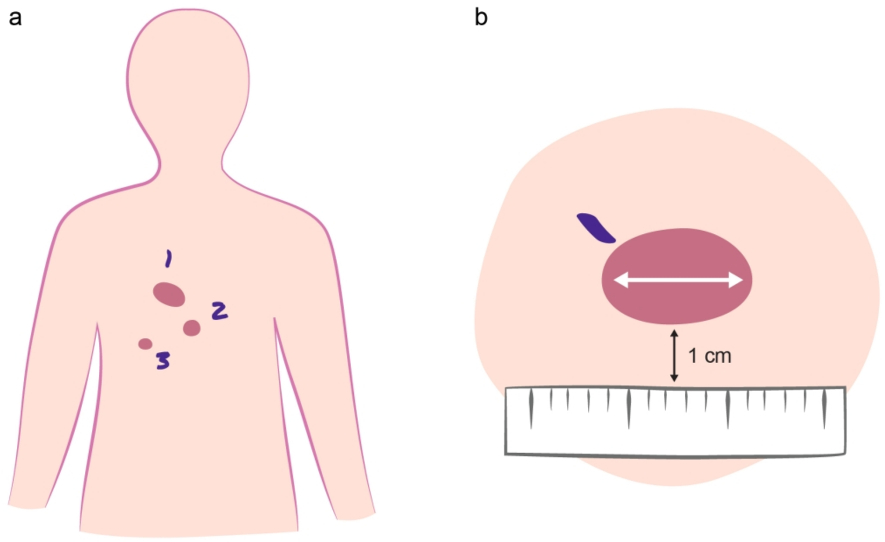

FIGURE 2
Pretreatment and follow up photography
Guide to overview photo of numbered target lesions (a) and photo of target lesion centrally in the image with longest diameter horizontally. Tumour marked proximally (violet) and adhesive ruler for scale 1 cm below the tumour at the bottom of the image (b). Tumours are numbered according to clinician preference, preferably with the most symptomatic tumour marked as tumour 1.

170x114mm (300 x 300 DPI)

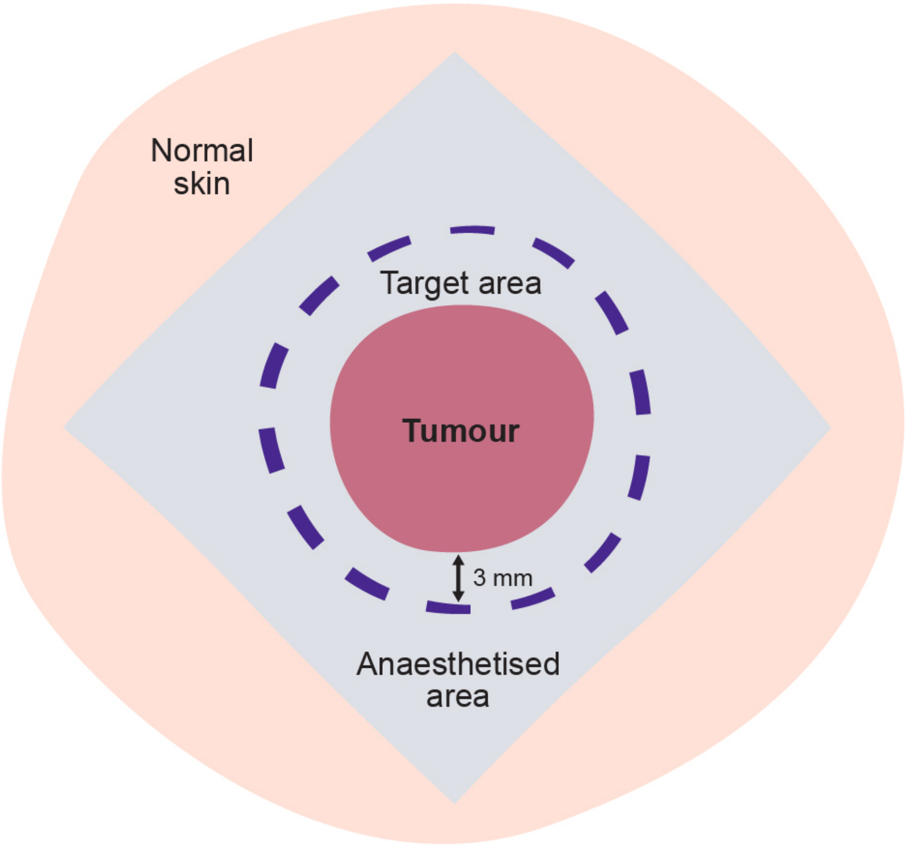

FIGURE 3
Treatment area
Local anaesthetic applied peri-tumourally in a rhomboid manner in an appropriate distance from the target area. For a total tumour treatment, the aim is to treat all the tumour with a 3 mm margin of clinically normal skin included in the target area. Blue-grey: anaesthetized area; Rose: tumour; Violet: Target margin.

87x84mm (300 x 300 DPI)

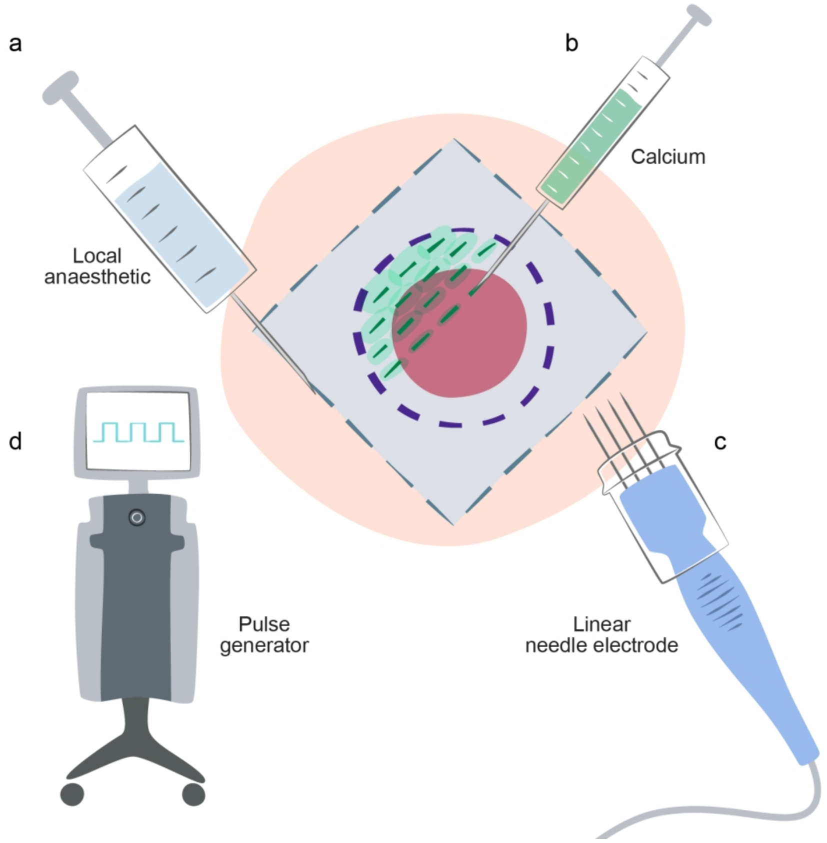

FIGURE 4
Calcium electroporation equipment
Local anaesthetic is injected around the target area with 10 ml syringes (a). Calcium is injected to the target area with 1 ml syringes (tumours under 3 cm in largest diameter) (b). For larger tumours syringes 3-5 ml may be appropriate. Pulse treatment is applied using linear electrodes (c) connected to a pulse generator (d) with a foot paddle that allows arming the electrode and delivery of the electrical pulses (e). Pulse parameters and delivered pulses are shown on the generator screen (f).

170x146mm (300 x 300 DPI)

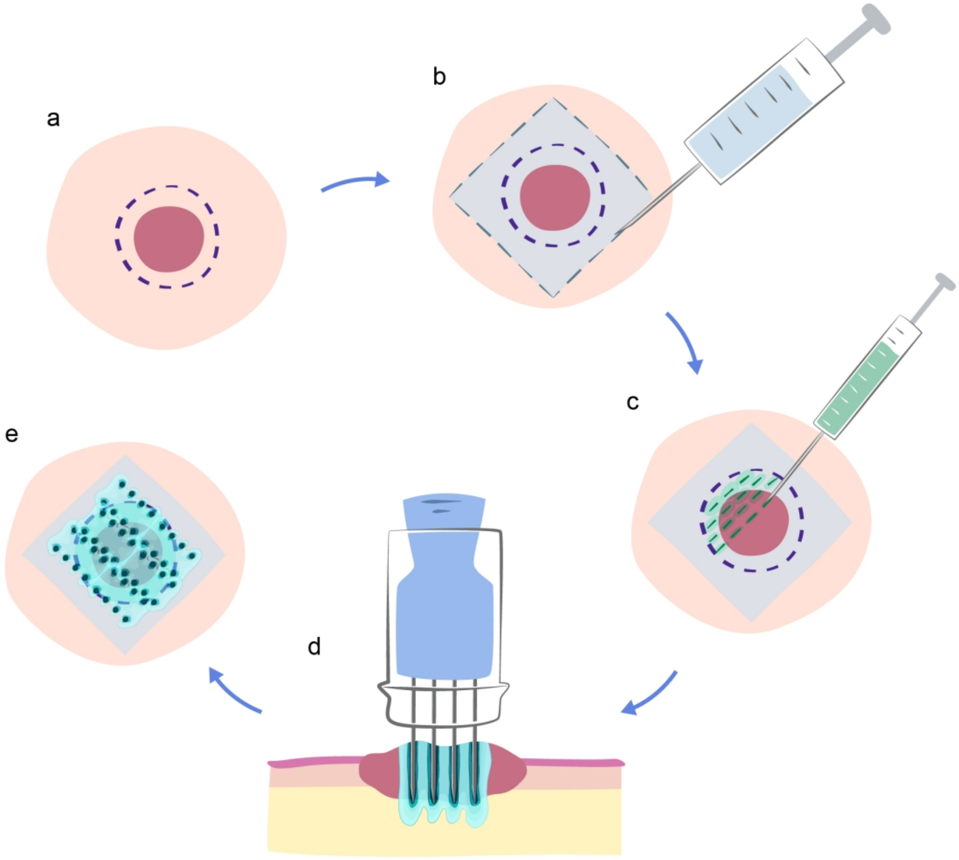

FIGURE 5
Calcium electroporation procedure
Local anaesthetic is injected dermally around the tumour in a rhomboid manner with an appropriate distance from the target area (a). The calculated i.t. dose of calcium is injected into the tumour in a parallel fashion throughout the tumour. The margin area is then supplemented with calcium until the calcium is evenly distributed throughout the entire target area (b). The electrode is applied into the target area (c). Max treatment depth is 3 cm and the needles should reach through the entire tumour depth and margin tissue below if possible. Electrode application (c) is repeated in an adjacent manner until the entire target area is treated (d). If multiple tumours are to be treated all targets are anaesthetized (a) first after which (b-d) is repeated for each target.

170x157mm (300 x 300 DPI)

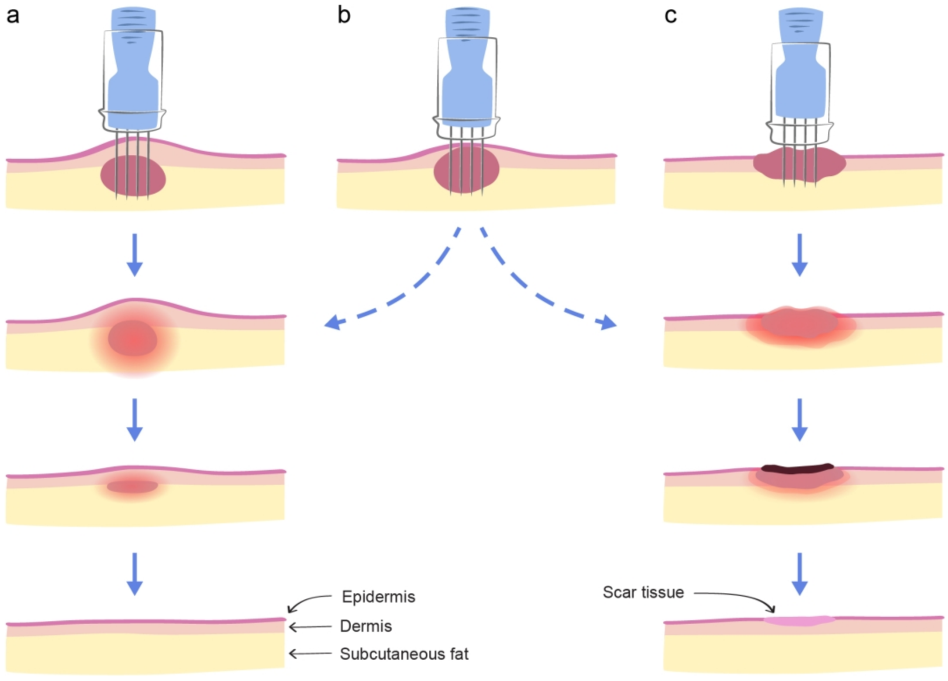

FIGURE 6
Cancer location in skin layers. Tumours may present as subcutaneous or cutaneous and involve some or all skin layers e.g. subcutaneous fat (a), dermis (b) and/or epidermis (c). If the epidermis is involved the tumours may present as ulcerating or fungating wounds.
Intact skin vs necrosis after CaEP. In most cases, subcutaneous tumours (a) will not ulcerate after calcium electroporation and skin will often appear intact after treatment. If treated tumours are situated as in (b) there may or may not be ulceration after treatment, depending on many factors such as patient healing potential and degree of invasion of the upper skin layers. Ulcerating or fungating tumours (c) may develop a necrotic, often crusted wound following treatment.

170x118mm (300 x 300 DPI)

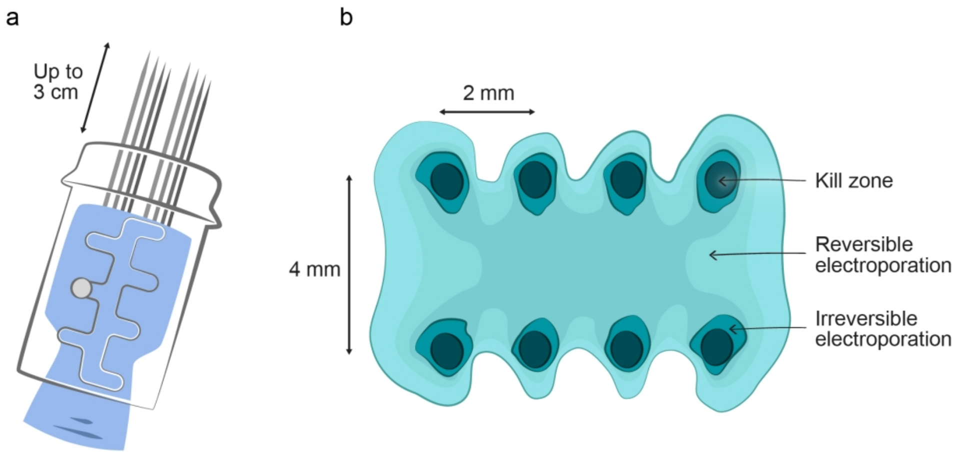

FIGURE 7
Distrubution of electric field
Linear array electrode for electroporation with 0.7 mm width 3 cm needles (IGEA) (a). Calculated E-field distribution at an applied voltage of 1.2 kV/cm for needle electrodes with two arrays of each four needles placed 4 mm apart (b). The spacing between the needles in the array is 2 mm. The small non-symmetric contour lines are due to limitations in the boundary conditions of the program, from Gehl et al.46 Note that inoculation of living cancer cells during treatment may be avoided by irreversible electroporation (>1.2 kV/cm) and cell-kill zone of tissue adjacent to the electrodes (>1.6 kV/cm).

170x86mm (300 x 300 DPI)

BMJ Open

Study protocol designed to investigate tumour response to calcium electroporation in cancers affecting the skin – a non-randomized phase II clinical trial

Journal:	*BMJ Open*
Manuscript ID	bmjopen-2020-046779.R1
Article Type:	Protocol
Date Submitted by the Author:	12-Mar-2021
Complete List of Authors:	Vissing, Mille; Sjællands Universitetshospital Roskilde, Department of Clinical Oncology and Palliative Care; University of Copenhagen, Department of Clinical Medicine<br>Ploen, John; University Hospital of Southern Denmark, Department of Oncology<br>Pervan, Mascha; University Hospital Schleswig Holstein, Department of Obstetrics and Gynecology<br>Vestergaard, Kitt; University College Absalon Campus Slagelse<br>Schnefeldt, Mazen; University Hospital of Southern Denmark, Department of Radiology<br>Frandsen, Stine; Sjællands Universitetshospital Roskilde, Department of Clinical Oncology and Palliative Care<br>Rafaelsen, Soeren; University Hospital of Southern Denmark, Department of Radiology<br>Lindhardt, Christina; University College Absalon Campus Soro; University of Southern Denmark, Clinical Institute<br>Jensen, Lars Henrik; University Hospital of Southern Denmark, Department of Oncology<br>Rody, Achim; University Hospital Schleswig Holstein, Department of Obstetrics and Gynecology<br>Gehl, Julie; Sjællands Universitetshospital Roskilde, Department of Clinical Oncology and Palliative Care; University of Copenhagen, Department of Clinical Medicine
**Primary Subject Heading**:	Oncology
Secondary Subject Heading:	Palliative care
Keywords:	ONCOLOGY, Dermatological tumours < ONCOLOGY, Adult oncology < ONCOLOGY, Adult palliative care < PALLIATIVE CARE, Breast tumours < ONCOLOGY

SCHOLARONE™
Manuscripts

I, the Submitting Author has the right to grant and does grant on behalf of all authors of the Work (as defined in the below author licence), an exclusive licence and/or a non-exclusive licence for contributions from authors who are: i) UK Crown employees; ii) where BMJ has agreed a CC-BY licence shall apply, and/or iii) in accordance with the terms applicable for US Federal Government officers or employees acting as part of their official duties; on a worldwide, perpetual, irrevocable, royalty-free basis to BMJ Publishing Group Ltd ("BMJ") its licensees and where the relevant Journal is co-owned by BMJ to the co-owners of the Journal, to publish the Work in this journal and any other BMJ products and to exploit all rights, as set out in our licence.

The Submitting Author accepts and understands that any supply made under these terms is made by BMJ to the Submitting Author unless you are acting as an employee on behalf of your employer or a postgraduate student of an affiliated institution which is paying any applicable article publishing charge ("APC") for Open Access articles. Where the Submitting Author wishes to make the Work available on an Open Access basis (and intends to pay the relevant APC), the terms of reuse of such Open Access shall be governed by a Creative Commons licence – details of these licences and which Creative Commons licence will apply to this Work are set out in our licence referred to above.

Other than as permitted in any relevant BMJ Author's Self Archiving Policies, I confirm this Work has not been accepted for publication elsewhere, is not being considered for publication elsewhere and does not duplicate material already published. I confirm all authors consent to publication of this Work and authorise the granting of this licence.

For peer review only

Study protocol designed to investigate tumour response to calcium electroporation in cancers affecting the skin – a non-randomized phase II clinical trial

Mille Vissing (1,2), John Ploen (3), Mascha Pervan (4), Kitt Vestergaard (5), Mazen Schnefeldt (6), Stine K Frandsen (1), Søren Rafael Rafaelsen (6), Christina Louise Lindhardt (5,7), Lars Henrik Jensen (3), Achim Rody (4), Julie Gehl* (1,2)

1. Center for Experimental Drug and Gene Electrotransfer (C*EDGE), Department of Clinical Oncology and Palliative Care, Zealand University Hospital, Roskilde and Næstved, Sygehusvej 10, 4000 Roskilde, Denmark
2. Department of Clinical Medicine, Faculty of Health and Medical Sciences, University of Copenhagen, Blegdamsvej 3B, 2200 Copenhagen, Denmark
3. Department of Oncology, University Hospital of Southern Denmark, Vejle, Beriderbakken 4, 7100 Vejle, Denmark
4. Department of Obstetrics and Gynecology, University Hospital Schleswig-Holstein, Campus Lübeck, Ratzeburger Allee 160, 23562 Lübeck, Germany
5. University College Absalon, Slagelsevej 7, 4180 Sorø, Denmark
6. Department of Radiology, University Hospital of Southern Denmark, Vejle, Beriderbakken 4, 7100 Vejle, Denmark
7. Clinical Institute, University of Southern Denmark, Windsløwparken 19, 5000 Odense C, Denmark

*Corresponding author: Prof. Julie Gehl, Center for Experimental Drug and Gene Electrotransfer (C*EDGE). Department of Clinical Oncology and Palliative Care, Zealand University Hospital, Sygehusvej 10, 4000 Roskilde, Denmark. E-mail: kgeh@regionsjaelland.dk.

For peer review only - http://bmjopen.bmj.com/site/about/guidelines.xhtml

Abstract

Introduction

Skin malignancy is a distressing problem for many patients and clinical management is challenging. This article describes the protocol for the Calcium Electroporation Response Study (CaEP-R) designed to investigate tumour response to calcium electroporation and is a descriptive guide to calcium electroporation treatment of malignant tumours in the skin. Calcium electroporation is a local treatment which induces supra-physiological intracellular calcium levels by intra-tumoural calcium administration and application of electrical pulses. The pulses create transient membrane pores allowing diffusion of non-permeant calcium ions into target cells. High calcium levels can kill cancer cells whilst normal cells can restore homeostasis. Prior trials with smaller cohorts have found calcium electroporation to be safe and efficient. This trial aims to include a larger multi-regional cohort of patients with different cancer diagnoses, and also to investigate treatment areas using magnetic resonance imaging (MRI) as well as assess impact on quality of life.

Methods and analysis

This non-randomized phase II multi-centre study will investigate response to calcium electroporation in 30 patients with cutaneous or subcutaneous malignancy. Enrolment of 10 patients is planned at three centres: Zealand University Hospital, University Hospital of Southern Denmark and University Hospital Schleswig-Holstein. Response after two months was chosen as the primary endpoint based on short-term response rates observed in a prior clinical study. Secondary endpoints include response to treatment using MRI and change in quality of life assessed by questionnaires and qualitative interviews.

Ethics and dissemination

The trial is approved by the Danish Medicines Agency and The Danish Regional Committee on Health Research Ethics. All included patients will receive active treatment (calcium electroporation). Patients can

For peer review only - http://bmjopen.bmj.com/site/about/guidelines.xhtml

continue systemic treatment during the study and side effects are expected to be limited. Data will be published in a peer-reviewed journal and made available to the public.

Trial registration

Trial registration: ClinicalTrials.gov, NCT04225767. Registered 13 January 2020.

EudraCT no: 2019-004314-34.

CaEP-R is part of Changing Cancer Care supported by the European Fund for Regional Development.

For peer review only

Strengths and limitations of this study

Strengths:

- This study investigates calcium electroporation as a novel treatment for cutaneous tumours.
- Information regarding different types of cancer will be obtained by including patients with tumours of any cancer histology.
- Procedures for calcium electroporation are clearly defined and tested in a multicentre setting.
- Calcium electroporation methods are thoroughly described in this protocol to facilitate clinical standardization.

Limitations:

- This study is limited in size (30 patients).

Keywords

Calcium, electroporation, skin, cancer, metastases, tumour response, MRI, qualitative interviews.

For peer review only - http://bmjopen.bmj.com/site/about/guidelines.xhtml

Introduction

Clinical challenges of cancer affecting the skin

Skin manifestation of malignancy is a distressing problem for a significant number of patients with cancer. The area of disease can vary in size from a few millimetres to extensive areas of the body, and surgical or oncological management is often challenging. The tumours can develop in the last few months of life but some are present for years and often require palliative management.[1] Calcium electroporation (CaEP) is a new, promising local treatment for cutaneous and subcutaneous malignancy.[2 3]

The following describes anti-cancer properties of calcium overloading using the electroporation technique and outlines preclinical discoveries as well as the first clinical experiences with calcium electroporation for different types of solid tumours, before describing protocol design.

Electroporation can be used to induce calcium overload

Electroporation is a method that can locally increase the intracellular concentration of otherwise non-permeant molecules. Application of short high-voltage pulses can create transient pores in cell membranes allowing molecules to diffuse in and out of targeted cells. The electroporation method can be used to increase the uptake of anti-neoplastic agents [4-7], nucleotides (i.e. DNA/RNA) [8-10] as well as non-permeant electrolytes such as calcium.[11-13]

Effects on normal and malignant cells - Preclinical studies

Calcium is a non-permeant ion involved in various cellular signalling processes such as gene transcription, proliferation, metabolism and apoptosis. Human eukaryote cells can restore calcium homeostasis by storing intracellular Ca^{2+} in cellular compartments, chelating Ca^{2+} to intracellular proteins and extruding Ca^{2+} from the cytosol (Figure 1).

Tumourigenesis alters cell calcium homeostasis through up- and down-regulation of calcium pumps and calcium channels, calcium storage compartments as well as changes in the cytoskeleton and membrane repair.[14] As a result, induction of cell death in cancer cells can be observed following calcium overload

For peer review only - http://bmjopen.bmj.com/site/about/guidelines.xhtml

(Figure 1).[15-18] Mitochondrial collapse, ATP depletion, and ultimately promotion of necrotic cell death, have been observed in cancer cells following CaEP *in vitro* and *in vivo.*[11 12 19] A preclinical cell study demonstrated that increased extracellular calcium alone does not influence cells in terms of decrease of ATP and cell death.[11 12]

Preclinical studies have shown that CaEP is efficient in inducing cell death *in vitro* - and *in vivo* across several cancer histologies including human breast-, bladder-, lung- and colon cancer cell lines.[11-13 20 21] Normal cells and tissues have been observed to be significantly more resistant to electroporation treatment than cancer cells and tissues in structured models.[13 21 22]

Calcium electroporation follows the principles of electrochemotherapy

Electrochemotherapy (ECT) is a standard therapy for cutaneous metastases where electroporation is used locally to increase cellular uptake of bleomycin, an anti-neoplastic drug.[23 24] ECT is well tested in treatment of different malignancies with both systemic- and local administration of bleomycin. Bleomycin induces apoptosis in cancer cells when delivered by electroporation and although the mechanisms through which calcium overload induces cell death differs from that of bleomycin (e.g. necrosis), the clinical setup and pulse parameters used for ECT are applicable.[2] CaEP is a local treatment where calcium is administered intra-tumourally after which electrical pulses are applied to the target area. Like ECT, calcium electroporation can be used for treatment of primary or secondary skin tumours or ulcerating malignant wounds such as basal and squamous cell skin cancers, melanoma skin cancer, Kaposi's sarcoma, breast cancer skin metastasis or head-and-neck cancer recurrence.[2 7 25-27] The procedure is performed in either general or local anaesthesia depending on tumour location and size, as well as patient preference and institutional operating procedure.[28 29]

For peer review only - http://bmjopen.bmj.com/site/about/guidelines.xhtml

Calcium electroporation for tumours affecting the skin and mucosa – Clinical studies

The first small clinical trial using calcium electroporation was designed as a double-blinded randomized study comparing electrochemotherapy and calcium electroporation.[2] This study showed CaEP to be safe, efficient and non-inferior to electrochemotherapy.

The trial included seven patients of which six had breast cancer and one had malignant melanoma. A total of 47 cutaneous metastases were treated of which 37 were randomized and evaluated for response and 10 were biopsied. A complete response (CR) was obtained in 66% (12/18) of calcium electroporated metastases versus 68% (13/19) of metastases treated with ECT.[2]

A second study with similar pre-set criteria, included seven patients with cutaneous metastasis (six patients with malignant melanoma and one with breast cancer). The study supported the safety and effectiveness of CaEP. While a difference in rates of CR was observed between metastases treated with CaEP (22%) and ECT (40%), both rates were lower compared to the first small initial trial.[2 3]

Finally, CaEP was proven to be safe and efficient in a trial investigating CaEP for recurrent head and neck cancer (n = 6) with a local response rate in treated lesions of 50% which is comparable to those of ECT. CaEP had limited side effects and one of the treated patients exhibited CR.[2 27]

Purpose of this study

The first clinical trials have shown encouraging results[2 3]. The purpose of this study is to investigate calcium electroporation as an anti-cancer treatment in a larger cohort to aid the translation of this easily implemented treatment to a standardised clinical setting. This study will also encompass response evaluation by magnetic resonance imaging (MRI) and quality of life (QoL) analyses.

Methods and Analysis

Design

This is a non-randomized, single-arm phase II trial. All patients will receive treatment and calcium electroporation will not be compared to other treatment modalities.

For peer review only - http://bmjopen.bmj.com/site/about/guidelines.xhtml

Setting

This study will be carried out at three cancer centres in Northern Europe: Zealand University Hospital, Denmark; University Hospital of Southern Denmark and University Hospital Schleswig-Holstein, Germany. The three year study period began February 11th 2020.

Participants

We seek to include 30 patients (10 at each site) with cutaneous malignancy of any type. Inclusion criteria in the trial are: 1) age older than 18 years of age; 2) ability to understand the participant information; 3) histologically verified cutaneous or subcutaneous cancer of any histology; 4) previously offered other relevant standard treatment for their cancer disease; 5) progressive or stable disease is present after a medical treatment period of two months or more (endocrine therapy, chemotherapy, immunotherapy etc.); 6) current radiation therapy does not involve the treated area; 7) performance status ECOG/WHO ≤2; 8) at least one cutaneous or subcutaneous tumour measuring up to 3 cm; 9) if sexually active, use of safe contraception (contraceptive coil, deposit injection of gestagen, subdermal implantation, hormonal vaginal ring or transdermal patch); 10) signed informed consent.

Exclusion criteria are pregnancy or lactation. Trial subjects are withdrawn from the study if the patient withdraws his or her consent; if disease progression requires new treatment strategies; investigator deems that withdrawing is in the best interest of the patient.

Primary endpoint

The primary endpoint is to evaluate the clinical response rate of calcium electroporation treatment of malignant tumours of the skin at two months after treatment. Response rate will be defined as number of responding lesions (partial or complete response) proportional to number of treated lesions, evaluated by changes in size (mm) clinically measured using a Vernier calliper. Tumour response will be documented

For peer review only - http://bmjopen.bmj.com/site/about/guidelines.xhtml

using clinical photography. A maximum of seven tumours up to 3 cm in largest diameter will be treated and followed per patient.

Response will be evaluated according to the modified RECIST guideline[30] and defined as: Complete Response (CR) – disappearance of the lesion; Partial Response (PR) – at least 30% decrease in the largest diameter of the lesion; Progressive Disease (PD) – at least 20% increase in the largest diameter of the lesion; Stable Disease (SD) – neither 30% decrease nor 20% increase of the largest diameter of the lesion.

Secondary endpoints

The secondary endpoints include: (1) Treatment response at month 1, 3, 4, 6 and 12; (2) Tumour and surrounding tissue histopathological regressive changes (e.g. % tumour cells and fibrosis) assessed by microscopy of biopsies taken from the treated area after 1 year; (3) Response after treatment on MRI scans on a subset of patients before and immediately after treatment, as well as after 2 months using diffusion-weighted magnetic resonance imaging (DW-MRI) as a method to monitor electroporated tissue, using the apparent diffusion coefficient (ADC); (4) QoL before and after treatment using European Organisation for Research and Treatment of Cancer (EORTC) Questionnaires; (5) QoL before treatment, after 2 months and after 1 year through EORTC QLQ-C15-PAL Core questionnaires evaluating cancer-related symptoms on a scale from 1-4 (not at all-very much) as well as overall QoL on a scale from 1-7 (very poor-excellent); (6) Observable systemic immunologic response from any routine scans (MRI, PET-CT etc.) before and after treatment in the inclusion period by tumour size and TNM stage; (7) Response rates and response duration according to tumour histology; (8) Complete and partial remissions of all treated tumours (defined as number of partially- or complete responding lesions relatively); (9) Rate of response for each individual patient; (10) Response (overall, as well as complete and partial) depending whether the treated tumour was in a previously irradiated area; (11) Current during treatment as measured by the pulse generator; (12) Qualitative interviews (in a subset of patients) performed before and 2 months after treatment which include measures related to patient experience and impact on QoL.

For peer review only - http://bmjopen.bmj.com/site/about/guidelines.xhtml

Ethics, safety and dissemination

The trial will be conducted in accordance with the official version of the Declaration of Helsinki and in agreement with The International Council for Harmonisation of Technical Requirements for Pharmaceuticals for Human Use (ICH) directions for Good Clinical Practice and the respective rules and regulations in Denmark and Germany.

As the treatment is safe and has not led to disease progression or increased tumour growth compared to controls in any preclinical or clinical studies, we expect CaEP treatment to be safe in treatment of cutaneous or subcutaneous malignant tumours of any histology.[2 3 11 13 21 22 25 26 31 32] Adverse Events (AE) and Serious Adverse Events (SAE) will be evaluated and graded according to CTCAE version 4.0. In view of the severity of metastatic cancer disease, there are certain conditions defined as SAEs but not reported as such in this study, e.g. voluntary hospitalization and surgery as treatment of the underlying cancer.

The trial is approved by the European Medicines Agency and Danish Medicines Agency and pending approval from relevant authorities in Germany. The trial is approved by the Danish Regional Committee on Health Research Ethics (Den Videnskabsetiske Komite for Region Sjælland), December 13, 2019 (case no: SJ-810), Data Protection Agency (no. REG-115-2019) and registered on EudraCT (no. 2019-004314-34) and ClinicalTrials.gov (Identifier: NCT04225767). Participation in the study requires signed informed consent. The study started on Febuar 11th 2020 and the first patient was included in the study on February 18th, 2020. Data will be published in a peer-reviewed journal. De-identified participant data are available from the corresponding author upon reasonable request. Reuse of data requires approval from the pertinent ethics committee. The results of the trial will be made available to the public by open access publication followed up with summaries posted on institution websites and other publically accessible sources.

For peer review only - http://bmjopen.bmj.com/site/about/guidelines.xhtml

Patient and Public Involvement

Patients and the public were not involved in the writing of the protocol in this study. A patient and public research panel will be engaged in the discussion of the outcomes of both response to therapy and the quality of life analysis.

Study Process

Preoperative assessment

After consent and inclusion in the study, data concerning medical history including cancer diagnosis, TNM classification, past- and ongoing treatments, demographic data, comorbidity, radiological data, pathology, clinical photos, electrocardiograms, blood tests (including haemoglobin, blood cell count, serum-calcium and C-reactive protein) and list of prescription medicine, will be recorded. Relevant data will be stored on the case report form and on a clinical trial management system (EasyTrial ApS, Aalborg, Denmark).

Definition of treatment target and photography

The visible and/or palpable target area is assessed clinically and marked (Figure 2).

The treated areas will be photographed prior to treatment on day 0 and at follow up 1, 2, 3, 4, 6 and 12 months after treatment. At each visit, an overview photo of the treatment sites will be taken, as well as photos of each target tumour (Figure 2).

Quality of Life assessment

Patient QoL is planned for evaluation before treatment, after two months and after one year, through using the EORTC certified QLQ-C15-PAL Core Questionnaire. A subset of patients treated at Zealand University Hospital will participate in a qualitative interview at month 0 (before treatment), 2 and 12, in order to assess treatment impact on the QoL of cancer patients treated with CaEP. The qualitative interview seeks to explore patients' physical and emotional well-being before and after treatment as well as expectations and experience

For peer review only - http://bmjopen.bmj.com/site/about/guidelines.xhtml

in relation to treatment. The interviews will be conducted as semi-structured face-to-face interviews as well as phone calls, both based on interview guides.[33] The recorded interviews will be transcribed and analysed to describe physical and emotional well-being experienced by the patients.

Anaesthesia

Paracetamol 1 g and/or lidocaine cream may be prescribed 1 hour preoperatively as a prophylactic analgesic for mild pain. Calcium electroporation will be performed using local anaesthesia with injection of lidocaine-epinephrine 1% peri-tumourally, although other anaesthesia may be used. A sufficient amount of local anaesthetics will be applied, which is comparative to other small local surgical procedures (Figure 3).
The maximum recommended dose of local anaesthesia will not be exceeded (30 ml for lidocaine-epinephrine 1%). The procedure may be performed in general anaesthesia and muscular relaxant administered, according to standard procedure of the institute. The equipment used in the CaEP procedure is described in Figure 4.

Calcium dose and tumour volume

Participants will be given a maximum of 20 ml calcium solution of 220 mmol/L administered intra-tumourally, equalling a total calcium dose of 4.4 mmol. The calcium dose in this trial will be analogous to the first phase II trials testing calcium electroporation on small cutaneous tumours.[2 3] Ten ml calcium chloride 500 mM is suspended in 12.7 ml isotonic NaCl solution to a calcium dose of 220 mmol/L. Mixing is performed bedside and countersigned by an observer. The dose to tumour volume ratio is an adapted calculation from the European Standard Operating Procedure of Electrochemotherapy (ESOPE) guidelines:[23 29] (1) tumour with a diameter <0.5 cm: 1 ml/cm^3 tumour tissue; (2) with a diameter from 0.5 cm to 3 cm: 0.5 ml/cm^3 of tumour tissue.
Tumour volume will be calculated according to ESOPE by the following formula: $V = ab^2 \pi / 6$ (a = the longest tumour diameter in cm; b = the longest diameter perpendicular to a).

For peer review only - http://bmjopen.bmj.com/site/about/guidelines.xhtml

It is important that the entire tumour volume and surrounding tissue is treated, thus we have chosen to define a treatment margin of 3 mm around the tumour when injecting calcium in the electroporation treatment area (Figure 3). Calcium will be administered intra-tumourally by manual injection with linear application upon needle retirement spaced around 5 mm, to ensure an equal distribution of calcium in the tumour (Figure 5). As the volume of peripheral tissue is infinitely variable (Figure 6 a-c), adequate volume of injected calcium chloride should be individually assessed by the treating clinician.

For practical purposes, a calcium solution (220 mmol/L) dose corresponding to half of tumour volume (ml) is distributed in the margin area. Due to differences in tumour situation in the cutaneous and subcutaneous layers, standardised dose of the tumour margin area can be a challenge (Figure 6 a-c). The intra-tumourally-injected volume and total injected dose for each tumour is noted.

Assuming a standard whole body extracellular volume (ECV) of 15 L (dependent on patient size), and a total calcium concentration of 2.2-2.55 mmol/L of which approximately half (1.18-1.32mmol/L) is unbound and metabolically active, a normal extracellular store of calcium is ~33-35 mmol of which 18-20 mmol is unbound. [34] Administering a maximum dose of 4.4 mmol calcium under the circumstances described above would lead to an increase in total calcium concentration from 2.55 mmol/L to 2.66 mmol/L. Although total calcium concentrations would rise, the concentration of free unbound calcium is tightly regulated by a buffer system primarily controlled by albumin, ensuring homeostasis.[34] Free extracellular calcium concentrations can rise, under normal circumstances, to 1.4 mmol/L with no apparent symptoms. Symptoms of hypercalcemia may occur when free calcium exceeds 1.6 mmol/L and includes increased thirst, frequent urination and abdominal pain (CTCAE 4.0). As previously mentioned, we expect the injected calcium to act locally, with an insignificant systemic rise in unbound calcium as observed in a previous study 6 hours post CaEP for recurrent head and neck cancer, injecting up to 13 ml 225 mmol/ml $CaCl_2$ i.t.[27] The method is deemed safe with local injection of up to 360 mg $CaCl_2$ (or 40 ml $CaCl_2$ 220 mmol/L solution). Moreover, electroporation causes a local hypo-perfusion through vasoconstriction thus the injected calcium will linger in the electroporated area, trapped intracellularly as membranes reseal (Figure 1).[22]

For peer review only - http://bmjopen.bmj.com/site/about/guidelines.xhtml

Applied electric pulses

We use linear array needle electrodes, which are superior in treatment of smaller tumours in the skin, to plate and hexagonal needle electrodes.[35] Pulses will be administered immediately after calcium injection, repositioning the electrode in an adjacent fashion to ensure an even distribution of treatment throughout the tumour (Figure 5). The needle application leaves small barely visible punctuations of the skin further allowing the treating clinician to keep track of treatment. The distributed electric field of a linear array needle electrode is illustrated in Figure 7.

We use the CE-certified Cliniporator (IGEA, Carpi, Italy) square wave pulse generator for electroporation, which delivers a series of eight consecutive pulses of 0.1 msec each with an amplitude of 1 kV/cm and a frequency of 1 Hz. For the linear array electrodes used in this study, 400V are applied. It has been observed that the linear array electrode led to a higher response rate in small cutaneous tumours[35], which supports the use of this electrode. The maximum current delivered during treatment as measured by the pulse generator will be noted for each tumour.

MRI

On a subset of patients, MRI will be used to assess response before (from 30 min up to 24 hours) and immediately after treatment (within 30 min up to 4 hours), as well as after 2 months using diffusion-weighted magnetic resonance imaging (DW-MRI) as a method to monitor electroporated tissue, using the apparent diffusion coefficient (ADC). ADC is a measure of the magnitude of diffusion of water molecules within tissue, clinically calculated using DW-MRI and expressed in units of mm^2/s.[36-38]

Side effects

The side effects of calcium electroporation for cutaneous and subcutaneous metastases less than 3 cm in diameter have been described in previous studies.[2 3] When the electrical pulse is given, there is a short-term contraction of the underlying muscles. Patients treated under local anaesthesia can experience muscle contraction as mildly unpleasant. Patients under general anaesthesia will not register this contraction. The

For peer review only - http://bmjopen.bmj.com/site/about/guidelines.xhtml

electric pulses last less than a second, and muscle contractions last for only the period in which the electric pulses are given. Experiences from treating small tumours with electrochemotherapy and calcium electroporation show that the treated area can initially become erythematous and swollen, but symptoms usually fade within hours to days. The area can subsequently become necrotic but generally heals within 6-10 weeks. In a few cases, infection may occur in the treated area. In case of infection, the patient may require antibiotics according to local guidelines.[2 3]

The treated area will usually require a bandage the first few days postoperatively because of slight oozing. Any postoperative pain will be treated according to local guidelines. A doctor will control the treated area at follow-up.

Patients are encouraged to take prescribed medications before, during and after treatment to make results more translational to a real world setting.

Any anticoagulant medication is continued, as the minor blood spill in treatment of tumours up to 3 cm in diameter is expected to be manageable.

Evaluation of treatment response

Treatment response is assessed by clinical measurement as described above in the endpoint sections. If treated areas present crusted wounds, the crust and any residual tumour is measured and clinical responsiveness noted. To evaluate long-term response, punch biopsies (preferably 4 mm) will be taken from the centre and edge of the treated areas one year after treatment. Biopsies of the tumour area will be stored as formalin-fixated, paraffin-embedded tissue blocks. Analyses will assess tumour- and surrounding tissue response to treatment histological analyses (e.g. percent tumour cells in sample and fibrosis). Samples will not be taken before 12 months follow up.

Sign of systemic immunologic response will be investigated from routine scans (MRI, PET-CT etc.) before and after treatment in the inclusion period by monitoring tumour size and TNM stage. Response rates and response duration according to tumour histology will be compared as well as the rate of response for each individual patient.

For peer review only - http://bmjopen.bmj.com/site/about/guidelines.xhtml

Statistical power calculation and definition of cohort

This is a phase II non-controlled study. The primary endpoint is to evaluate tumour response (overall response which includes partial- and complete responses) two months after CaEP treatment evaluated by clinical examination and documented by photography. The appropriate sample size is calculated to determine the minimum number of subjects that need to be enrolled in this study, in order to have sufficient statistical power in detecting calcium electroporation treatment effect.

Based on the response rate from the initial, but small study with calcium electroporation treatment we predicted a mean complete response rate of 66% and an estimated SD of 36%.[2] In order to define our cohort, a response rate of no treatment is estimated to be 0%. To detect a 66% difference with a power of 90%, eight patients should be included. As we aim to support earlier studies in a real-world setting, 10 patients from each involved cancer centre will be included. Because our primary end point is follow-up after 2 months we cautiously predict that 2 patients out of 10 will not be evaluable for the primary endpoint. We thereby expect 24 evaluable patients at primary end point with 95% CI. Off-study patients will not lead to further inclusion.

At least one tumour will be treated in each patient (up to 7 tumours per patient may be included) and the response rate across all treated tumours will be calculated.

Discussion

Tumour response to calcium electroporation

This study seeks to illuminate the treatment effect of CaEP in a larger cohort of patients with cutaneous or subcutaneous malignancy of different histological features. As preclinical studies have shown response to treatment in all tested cancer cell-lines of different types,[12 13 20-22] clinical studies using CaEP for any solid tumours are warranted.

For peer review only - http://bmjopen.bmj.com/site/about/guidelines.xhtml

Conducting a phase II trial in a multicentre setting, operating and including at three cancer centres, presents the possibility of further investigating this novel treatment modality in a larger cohort. One team has previous experience with CaEP, another with ECT and one centre has no previous experience with electroporation treatment.

The multi-centre setting allows for investigating interesting endpoints in smaller subgroups (e.g. MRI and QoL) due to practical advantages and collaborations at the involved centres.

MRI for visualising calcium electroporation

In the planned study, the University Hospital of Southern Denmark will use MRI to visualise and quantify the immediate effects of CaEP in a subgroup of patients. This method could provide the first imaging of immediate skin tumour response to treatment with CaEP and assess the treated area perioperatively. The results of MRI may uncover whether cell swelling for CaEP can be visualized after treatment of malignant tumours of the skin, thus aiding postoperative verification of treatment area. Follow up MRI may also help visualize differences in healing outcomes and mechanisms in subcutaneous and cutaneous tumours respectively.

Although different cancer entities will represent a heterogeneous cohort, it will facilitate comparison of response rates and response duration according to tumour type. Furthermore, differences in response depending on whether the treated tumour was in a previously irradiated area will be analysed.

Qualitative interviews to asses treatment impact

The centre at Zealand University Hospital will use qualitative interviews in the first attempt to provide more detailed and nuanced data regarding QoL in patients treated with CaEP than standardised questionnaires.[39] Qualitative data may further uncover concerns and priorities that distinguish cancer patients with skin malignancy from other patient populations and help to adequately capture patients' experience.

For peer review only - http://bmjopen.bmj.com/site/about/guidelines.xhtml

Limitations

The proposed study is a clinical prospective study investigating a heterogeneous group of patients with malignancies at different disease stages. The included patients will vary in number and type of previous and ongoing therapies. The number of included patients is limited and no control group will be included. With expected inclusion of 30 patients, statistical conclusions will need to be drawn based on even smaller subgroups of tumour entities. Furthermore, the tumours to be included in this study will not have responded to standard therapies after two months and may represent a cohort of treatment-resistant cancers with lower response rates than in a real world setting.

The clinical significance of the effect of CaEP on QoL will be limited to patients with cutaneous metastases up to 3 cm in diameter. Stigmatising or distressing symptoms such as suppuration, malodour and pain, often correlate with more advanced skin tumours.

Perspectives

The response rates of metastatic disease could be important for future studies investigating calcium electroporation as a treatment modality for malignancy of internal organs. Using calcium electroporation as pre- or post-surgical treatment or combined with interventional radiology could be interesting to explore in future studies. Using calcium in procedures using irreversible electroporation, an established technique used for tissue ablation, could potentially improve outcomes in cancer surgery as it has been shown that there is a perimeter of tissue that undergoes reversible electroporation around ablated treatment areas.[40]

If supported, calcium electroporation will be a readily accessible, efficient treatment for appurtenant facilities equipped with electroporation equipment. Using calcium in electroporation treatments could benefit clinicians and patients alike, as calcium is a safe, naturally occurring electrolyte with low cost, easy handling and limited side effects.

For peer review only - http://bmjopen.bmj.com/site/about/guidelines.xhtml

List of abbreviations

EP: Electroporation; CaEP: Calcium electroporation; CaEP-R: Study title; $CaCl_2$: Calcium chloride; QoL: Quality of life; ECT: Electrochemotherapy, ECV: Extracellular volume; ATP: Adenosine triphosphate; CI: Confidence interval; SD: Standard deviation; EORTC: European Organisation for Research and Treatment of Cancer; AE: Adverse event; SAE: Serious adverse event; PR: Partial response; CR: Complete response; PD: Progressive disease; SD: Stable disease.

Authors' contributions

MV was the primary author of the protocol and the manuscript. The study concept was initially designed by MV and JG, expanded for MRI, QoL and execution in Germany by LHJ, JP, SRR, CLL, MP and AR. MV, SKF, KV, MS and JG contributed to writing of the manuscript. All authors read and approved the final manuscript.

Competing interests

JG and SKF are co-inventors of a patent regarding calcium electroporation.Therapeutic applications of calcium electroporation to effectively induce tumour necrosis. Granted. PCT/DK2012/050496

Funding

The Interreg consortium Changing Cancer Care (CCC) 094-1.1-18 funded by the European Regional Development Fund has supported the project.

Acknowledgements

Not applicable.

For peer review only - http://bmjopen.bmj.com/site/about/guidelines.xhtml

For peer review only

References

1. van Leeuwen BL, Houwerzijl M, Hoekstra HJ. Educational tips in the treatment of malignant ulcerating tumours of the skin. *Eur J Surg Oncol* 2000;26(5):506-8. doi: 10.1053/ejso.1999.0931 [published Online First: 2000/10/18]
2. Falk H, Matthiessen LW, Wooler G, et al. Calcium electroporation for treatment of cutaneous metastases; a randomized double-blinded phase II study, comparing the effect of calcium electroporation with electrochemotherapy. *Acta Oncol* 2018;57(3):311-19. doi: 10.1080/0284186X.2017.1355109 [published Online First: 2017/08/18]
3. Agoston D, Baltas E, Ocsai H, et al. Evaluation of Calcium Electroporation for the Treatment of Cutaneous Metastases: A Double Blinded Randomised Controlled Phase II Trial. *Cancers (Basel)* 2020;12(1) doi: 10.3390/cancers12010179 [published Online First: 2020/01/16]
4. Mir LM, Banoun H, Paoletti C. Introduction of definite amounts of nonpermeant molecules into living cells after electropermeabilization: direct access to the cytosol. *Experimental Cell Research* 1988;175(1):15-25.
5. Sersa G, Cemazar M, Miklavcic D. Antitumor effectiveness of electrochemotherapy with cis-diamminedichloroplatinum(II) in mice. *Cancer Research* 1995;55(15):3450-55.
6. Gothelf A, Mir LM, Gehl J. Electrochemotherapy: results of cancer treatment using enhanced delivery of bleomycin by electroporation. *Cancer Treatment Reviews* 2003;29(5):371-87.
7. Clover AJP, Salwa SP, Bourke MG, et al. Electrochemotherapy for the treatment of primary basal cell carcinoma; A randomised control trial comparing electrochemotherapy and surgery with five year follow up. *Eur J Surg Oncol* 2020;46(5):847-54. doi: 10.1016/j.ejso.2019.11.509 [published Online First: 2019/12/22]
8. Neumann E, Schaefer-Ridder M, Wang Y, et al. Gene transfer into mouse lyoma cells by electroporation in high electric fields. *EMBO J* 1982;1(7):841-45.
9. Heller L, Pottinger C, Jaroszeski MJ, et al. In vivo electroporation of plasmids encoding GM-CSF or interleukin-2 into existing B16 melanomas combined with electrochemotherapy induces long-term antitumour immunity. *Melanoma Res* 2000;10(0960-8931 SB - IM):577-83.
10. Sersa G, Teissie J, Cemazar M, et al. Electrochemotherapy of tumors as in situ vaccination boosted by immunogene electrotransfer. *Cancer Immunol Immunother* 2015;64(10):1315-27. doi: 10.1007/s00262-015-1724-2 [published Online First: 2015/06/13]
11. Frandsen SK, Gissel H, Hojman P, et al. Direct therapeutic applications of calcium electroporation to effectively induce tumor necrosis. *Cancer Res* 2012;72(6):1336-41. doi: 10.1158/0008-5472.CAN-11-3782 [published Online First: 2012/01/28]
12. Hansen EL, Sozer EB, Romeo S, et al. Dose-dependent ATP depletion and cancer cell death following calcium electroporation, relative effect of calcium concentration and electric field strength. *PLoS One* 2015;10(4):e0122973. doi: 10.1371/journal.pone.0122973
13. Frandsen SK, Kruger MB, Mangalanathan UM, et al. Normal and Malignant Cells Exhibit Differential Responses to Calcium Electroporation. *Cancer Res* 2017;77(16):4389-401. doi: 10.1158/0008-5472.CAN-16-1611 [published Online First: 2017/08/02]
14. Frandsen SK, Vissing M, Gehl J. A Comprehensive Review of Calcium Electroporation -A Novel Cancer Treatment Modality. *Cancers (Basel)* 2020;12(2) doi: 10.3390/cancers12020290 [published Online First: 2020/01/30]
15. Aung CS, Kruger WA, Poronnik P, et al. Plasma membrane Ca2+-ATPase expression during colon cancer cell line differentiation. *Biochem Biophys Res Commun* 2007;355(4):932-6. doi: 10.1016/j.bbrc.2007.02.050 [published Online First: 2007/02/27]

For peer review only - http://bmjopen.bmj.com/site/about/guidelines.xhtml

16. Papp B, Brouland JP, Arbabian A, et al. Endoplasmic reticulum calcium pumps and cancer cell differentiation. *Biomolecules* 2012;2(1):165-86. doi: 10.3390/biom2010165 [published Online First: 2012/01/01]
17. Rizzuto R, De Stefani D, Raffaello A, et al. Mitochondria as sensors and regulators of calcium signalling. *Nat Rev Mol Cell Biol* 2012;13(9):566-78. doi: 10.1038/nrm3412 [published Online First: 2012/08/02]
18. Bonora M, Bononi A, De Marchi E, et al. Role of the c subunit of the FO ATP synthase in mitochondrial permeability transition. *Cell Cycle* 2013;12(4):674-83. doi: 10.4161/cc.23599 [published Online First: 2013/01/25]
19. Gibot L, Montigny A, Baaziz H, et al. Calcium Delivery by Electroporation Induces In Vitro Cell Death through Mitochondrial Dysfunction without DNA Damages. *Cancers (Basel)* 2020;12(2) doi: 10.3390/cancers12020425 [published Online First: 2020/02/16]
20. Frandsen SK, Gissel H, Hojman P, et al. Calcium electroporation in three cell lines: a comparison of bleomycin and calcium, calcium compounds, and pulsing conditions. *Biochim Biophys Acta* 2014;1840(3):1204-8. doi: 10.1016/j.bbagen.2013.12.003 [published Online First: 2013/12/18]
21. Frandsen SK, Gibot L, Madi M, et al. Calcium Electroporation: Evidence for Differential Effects in Normal and Malignant Cell Lines, Evaluated in a 3D Spheroid Model. *PLoS One* 2015;10(12):e0144028. doi: 10.1371/journal.pone.0144028 [published Online First: 2015/12/04]
22. Staresinic B, Jesenko T, Kamensek U, et al. Effect of calcium electroporation on tumour vasculature. *Sci Rep* 2018;8(1):9412. doi: 10.1038/s41598-018-27728-z [published Online First: 2018/06/22]
23. Marty M, Sersa G, Garbay JR, et al. Electrochemotherapy - An easy, highly effective and safe treatment of cutaneous and subcutaneous metastases: Results of ESOPE (European Standard Operating Procedures of Electrochemotherapy) study. *Ejc Supplements* 2006;4(11):3-13.
24. Cadossi R, Ronchetti M, Cadossi M. Locally enhanced chemotherapy by electroporation: clinical experiences and perspective of use of electrochemotherapy. *Future Oncol* 2014;10(5):877-90. doi: 10.2217/fon.13.235
25. Falk H, Lambaa S, Johannesen HH, et al. Electrochemotherapy and calcium electroporation inducing a systemic immune response with local and distant remission of tumors in a patient with malignant melanoma - a case report. *Acta Oncol* 2017;56(8):1126-31. doi: 10.1080/0284186X.2017.1290274
26. Szewczyk A, Gehl J, Daczewska M, et al. Calcium electroporation for treatment of sarcoma in preclinical studies. *Oncotarget* 2018;9(14):11604-18. doi: 10.18632/oncotarget.24352 [published Online First: 2018/03/17]
27. Plaschke CC, Gehl J, Johannesen HH, et al. Calcium electroporation for recurrent head and neck cancer: A clinical phase I study. *Laryngoscope Investig Otolaryngol* 2019;4(1):49-56. doi: 10.1002/lio2.233 [published Online First: 2019/03/05]
28. Mir LM, Gehl J, Sersa G, et al. Standard operating procedures of the electrochemotherapy: Instructions for the use of bleomycin or cisplatin administered either systemically or locally and electric pulses delivered by the CliniporatorTM by means of invasive or non-invasive electrodes. *European Journal of Cancer Supplements* 2006;4(11):14-25.
29. Gehl J, Sersa G, Matthiessen LW, et al. Updated standard operating procedures for electrochemotherapy of cutaneous tumours and skin metastases. *Acta Oncol* 2018;57(7):874-82. doi: 10.1080/0284186X.2018.1454602 [published Online First: 2018/03/27]
30. Eisenhauer EA, Therasse P, Bogaerts J, et al. New response evaluation criteria in solid tumours: revised RECIST guideline (version 1.1). *European Journal of Cancer* 2009;45:228-47.
31. Hoejholt KL, Muzic T, Jensen SD, et al. Calcium electroporation and electrochemotherapy for cancer treatment: Importance of cell membrane composition investigated by lipidomics, calorimetry and in vitro efficacy. *Sci Rep* 2019;9(1):4758. doi: 10.1038/s41598-019-41188-z [published Online First: 2019/03/22]

For peer review only - http://bmjopen.bmj.com/site/about/guidelines.xhtml

32. Falk H, Forde PF, Bay ML, et al. Calcium electroporation induces tumor eradication, long-lasting immunity and cytokine responses in the CT26 colon cancer mouse model. *Oncoimmunology* 2017;6(5):e1301332. doi: 10.1080/2162402X.2017.1301332 [published Online First: 2017/06/24]
33. Glesne C. Becoming Qualitative Researchers. An Introduction. 5. ed: Pearson 2016.
34. Baird GS. Ionized calcium. *Clin Chim Acta* 2011;412(9-10):696-701. doi: 10.1016/j.cca.2011.01.004 [published Online First: 2011/01/18]
35. Clover J, Terlizzi Fd, Bertino G, et al. Electrochemotherapy in the treatment of cutaneous malignancy; outcomes and subgroup analysis from the cumulative results from the pan- European InspECT Database for 2482 lesions in 987 patients (2008-2019). *European Journal of Cancer* 2020
36. Sener RN. Diffusion MRI: apparent diffusion coefficient (ADC) values in the normal brain and a classification of brain disorders based on ADC values. *Comput Med Imaging Graph* 2001;25(4):299-326. doi: 10.1016/s0895-6111(00)00083-5 [published Online First: 2001/05/18]
37. Mahmood F, Hansen RH, Agerholm-Larsen B, et al. Diffusion-weighted MRI for verification of electroporation-based treatments. *J Membr Biol* 2011;240(3):131-8. doi: 10.1007/s00232-011-9351-0 [published Online First: 2011/03/08]
38. Mahmood F, Hansen RH, Agerholm-Larsen B, et al. Detection of electroporation-induced membrane permeabilization states in the brain using diffusion-weighted MRI. *Acta Oncol* 2015;54(3):289-97. doi: 10.3109/0284186X.2014.991045 [published Online First: 2015/01/17]
39. Bhat TS, Herbosa CM, Rosenberg AR, et al. Current measures are not sufficient: an interview-based qualitative assessment of quality of life in cutaneous T-cell lymphoma. *Br J Dermatol* 2020 doi: 10.1111/bjd.19298 [published Online First: 2020/06/09]
40. Hjouj M, Last D, Guez D, et al. MRI study on reversible and irreversible electroporation induced blood brain barrier disruption. *PLoS One* 2012;7(1932-6203 (Electronic)):e42817-e17.

FIGURE 1

Schematic overview of known effects of calcium electroporation on normal cells and cancer cells

Electroporation pulse treatment creates transient pores in both cancer cells (bottom row) and normal cells (top row). In a high calcium concentration environment, the intracellular calcium level is greatly increased immediately after treatment. After a few minutes, the plasma membrane reseals and both normal and malignant cells are overloaded with Ca^{2+}. Ca^{2+}-extrusion from the cytoplasm is carried out by Na^{+}-Ca^{2+} exchangers, plasma membrane Ca^{2+}-ATPase (PMCA) pumps and sarco-endoplasmic reticulum Ca^{2+}-ATPase (SERCA) pumps that transport Ca^{2+} into the extracellular space and into the endoplasmic reticulum (ER) lumen respectively, whilst consuming ATP in both normal and cancer cells. Suppression of free cytosolic calcium is further facilitated by buffering in mitochondrial compartments and binding to Ca^{2+}-binding proteins. Calcium pumps and channels may be up- or downregulated in malignant cells, impairing calcium homeostasis. Furthermore, cancer cells can have weakened membrane repair mechanisms. Ca^{2+} overload

For peer review only - http://bmjopen.bmj.com/site/about/guidelines.xhtml

induces mitochondrial dysfunction and critical ATP depletion in cancer cells. These properties may act as mechanisms of calcium electroporation-induced cell death. As illustrated, electroporation may also cause cellular swelling. Following calcium electroporation, normal cells have the ability to restore homeostasis while cancer cells are prone to necrotise. Cancer cell necrosis leads to release of a danger signal as well as cell remnants that may induce a local and/or systemic immune response.

FIGURE 2

Pretreatment and follow up photography

Guide to overview photo of numbered target lesions (a) and photo of target lesion centrally in the image with longest diameter horizontally. Tumour marked proximally (violet) and adhesive ruler for scale 1 cm below the tumour at the bottom of the image (b). Tumours are numbered according to clinician preference, preferably with the most symptomatic tumour marked as tumour 1.

FIGURE 3

Treatment area

Local anaesthetic applied peri-tumourally in a rhomboid manner in an appropriate distance from the target area. For a total tumour treatment, the aim is to treat all visible and/or palpable tumours with a 3 mm margin of clinically normal skin included in the target area. Blue-grey: anaesthetized area; Rose: tumour; Violet: Target margin.

FIGURE 4

Equipment for calcium electroporation

(a) Anesthesia is necessary, either local anesthesia or other (depending on tumour location and size). (b) Calcium is always administered by local injection, and using e.g. 1 ml syringes ensures easy and steady administration. (c) Electric pulse delivery is performed by needle electrodes that can penetrate so that the

For peer review only - http://bmjopen.bmj.com/site/about/guidelines.xhtml

bottom of the tumour can be covered. The linear array electrode is preferred due to superior results for smaller tumours. (d) A square wave pulse generator (electroporator) enables precise delivery of pulses of the planned treatment sequence, in this case 8 pulses of 0.1 ms with a voltage of 400 V (corresponding to 1 kV/cm applied voltage to electrode distance ratio, as used for the linear array electrode).

FIGURE 5

Calcium electroporation procedure

(a) The target area is defined as the area which is clinically visualized as tumour + a margin. (b) When performing the local anesthesia it is important to provide coverage of the margin as well as a zone around the margin so that the electrodes may be inserted without discomfort. Adding further local anesthetic below the tumour can also be helpful, in particular when treating larger lesions. (c) The calculated i.t. dose of calcium is injected into the tumour in a parallel fashion throughout the tumour. The margin area is then supplemented with calcium until the calcium is evenly distributed throughout the entire target area. (d) The electrode is inserted so that needles reach just beyond the deepest part of the tumour, and a pulse sequence is applied. The electrode can then be subsequently inserted in a systematic way to cover the entire tumour volume, as indicated in (e) As can be seen, the treatment area then covers the tumour with treatment margin.

FIGURE 6

Cancer location in skin layers

Tumours may present as subcutaneous or cutaneous and involve some or all skin layers e.g. subcutaneous fat (a), dermis (b) and/or epidermis (c). If the epidermis is involved the tumours may present as ulcerating or fungating wounds.

Intact skin vs necrosis after CaEP. In most cases, subcutaneous tumours (a) will not ulcerate after calcium electroporation and skin will often appear intact after treatment. If treated tumours are situated as in (b) there may or may not be ulceration after treatment, depending on many factors such as patient healing potential and degree of invasion of the upper skin layers. Ulcerating or fungating tumours (c) may develop a necrotic, often crusted wound following treatment.

For peer review only - http://bmjopen.bmj.com/site/about/guidelines.xhtml

FIGURE 7

Distribution of electric field

(a) The linear array needle electrodes have needles of 0.7 mm that can be extended up to 3 cm. (b) A diagram of field distribution adapted from Gehl et al (BBA 1999) shows that the distance between arrays is 4 mm, and distance between needles in the array app. 2 mm. At the needle insertion points, high fields will be present that may lead to irreversible electroporation. In the zone for reversible electroporation cell death will be due to effect of internalized calcium.

For peer review only

For peer review only - http://bmjopen.bmj.com/site/about/guidelines.xhtml

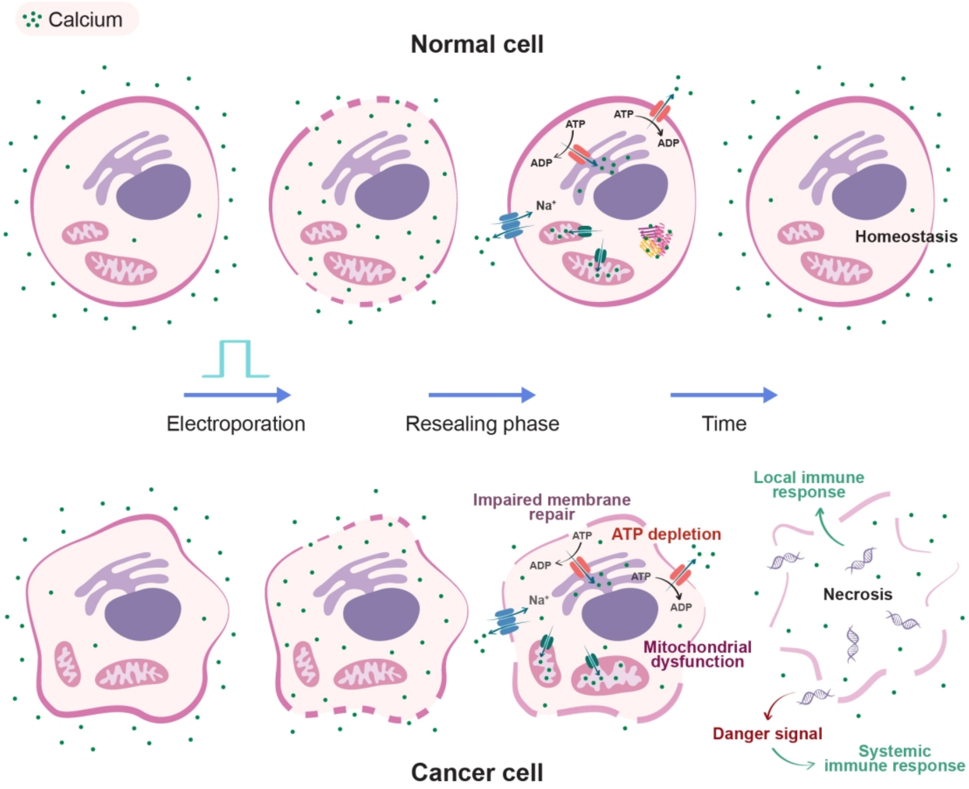

FIGURE 1

Schematic overview of known effects of calcium electroporation on normal cells and cancer cells

Electroporation pulse treatment creates transient pores in both cancer cells (bottom row) and normal cells (top row). In a high calcium concentration environment, the intracellular calcium level is greatly increased immediately after treatment.12 After a few minutes, the plasma membrane reseals and both normal and malignant cells are overloaded with Ca2+. Ca2+-extrusion from the cytoplasm is carried out by Na+-Ca2+ exchangers, plasma membrane Ca2+-ATPase (PMCA) pumps and sarco-endoplasmic reticulum Ca2+-ATPase (SERCA) pumps that transport Ca2+ into the extracellular space and into the endoplasmic reticulum (ER) lumen respectively, whilst consuming ATP in both normal and cancer cells.41 Suppression of free cytosolic calcium is further facilitated by buffering in mitochondrial compartments and binding to Ca2+-binding proteins. Calcium pumps and channels may be up- or downregulated in malignant cells, impairing calcium homeostasis.15 16 42 43 Furthermore, cancer cells can have weakened membrane repair mechanisms.44 Ca2+ overload induces mitochondrial dysfunction and critical ATP depletion in cancer cells.12 19 44 These properties may act as mechanisms of calcium electroporation-induced cell death. As illustrated, electroporation may also cause cellular swelling.45 Following calcium electroporation, normal cells have the ability to restore homeostasis while cancer cells are prone to necrotise. Cancer cell necrosis leads to release of a danger signal as well as cell remnants that may induce a local and/or systemic immune response.40

170x141mm (300 x 300 DPI)

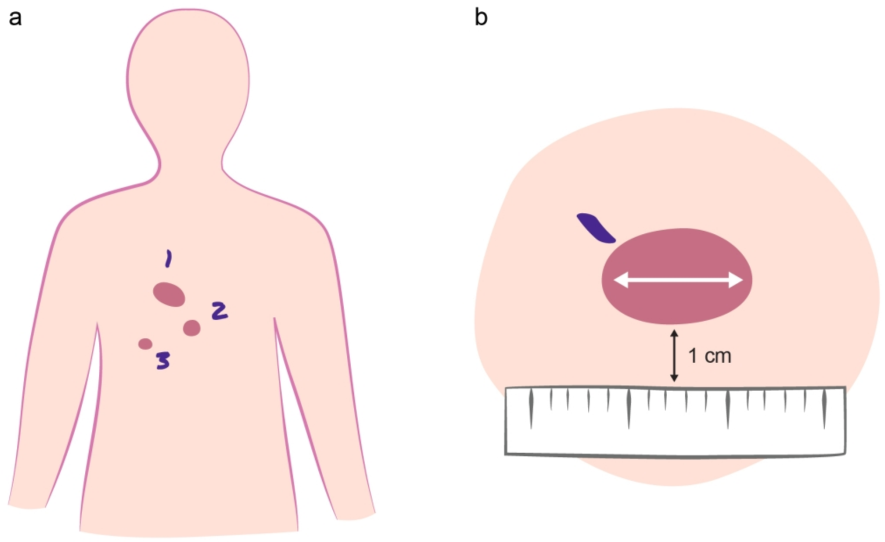

FIGURE 2
Pretreatment and follow up photography
Guide to overview photo of numbered target lesions (a) and photo of target lesion centrally in the image with longest diameter horizontally. Tumour marked proximally (violet) and adhesive ruler for scale 1 cm below the tumour at the bottom of the image (b). Tumours are numbered according to clinician preference, preferably with the most symptomatic tumour marked as tumour 1.

170x114mm (300 x 300 DPI)

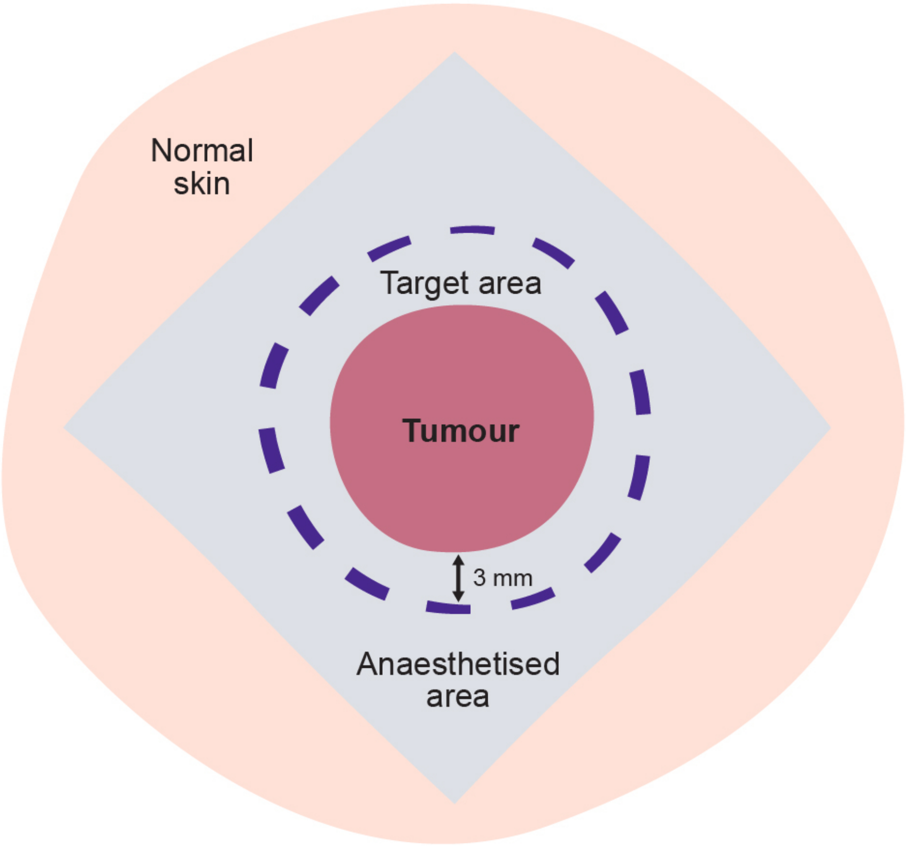

FIGURE 3
Treatment area
Local anaesthetic applied peri-tumourally in a rhomboid manner in an appropriate distance from the target area. For a total tumour treatment, the aim is to treat all visible and/or palpable tumours with a 3 mm margin of clinically normal skin included in the target area. Blue-grey: anaesthetized area; Rose: tumour; Violet: Target margin.

87x84mm (300 x 300 DPI)

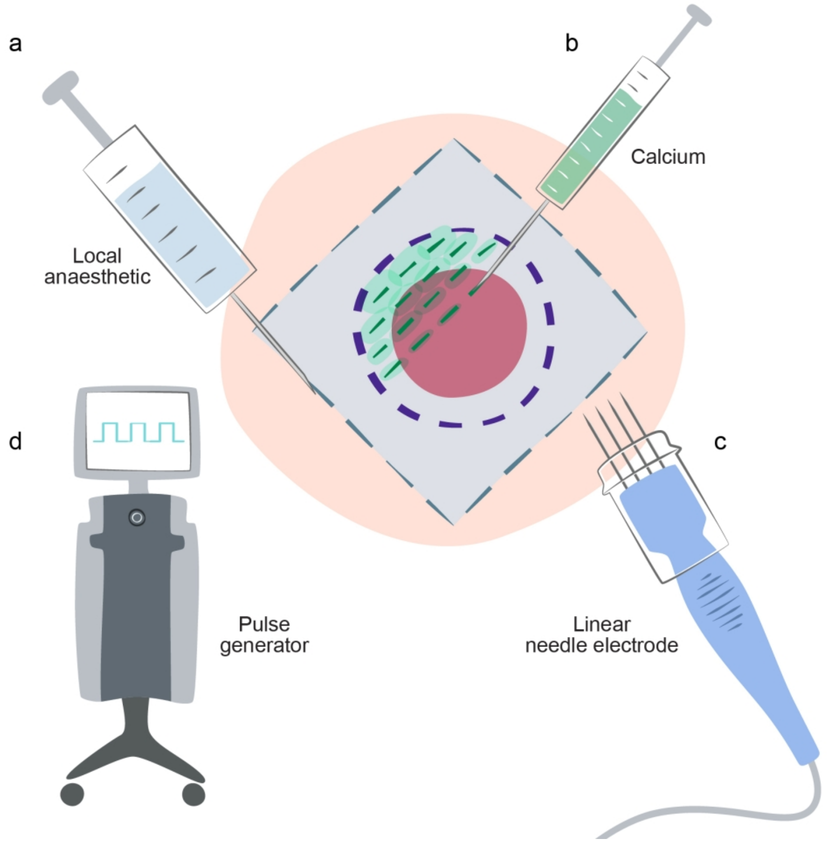

FIGURE 4
Equipment for calcium electroporation
(a) Anesthesia is necessary, either local anesthesia or other (depending on tumour location and size). (b) Calcium is always administered by local injection, and using e.g. 1 ml syringes ensures easy and steady administration. (c) Electric pulse delivery is performed by needle electrodes that can penetrate so that the bottom of the tumour can be covered. The linear array electrode is preferred due to superior results for smaller tumours. (d) A square wave pulse generator (electroporator) enables precise delivery of pulses of the planned treatment sequence, in this case 8 pulses of 0.1 ms with a voltage of 400 V (corresponding to 1 kV/cm applied voltage to electrode distance ratio, as used for the linear array electrode).

170x146mm (300 x 300 DPI)

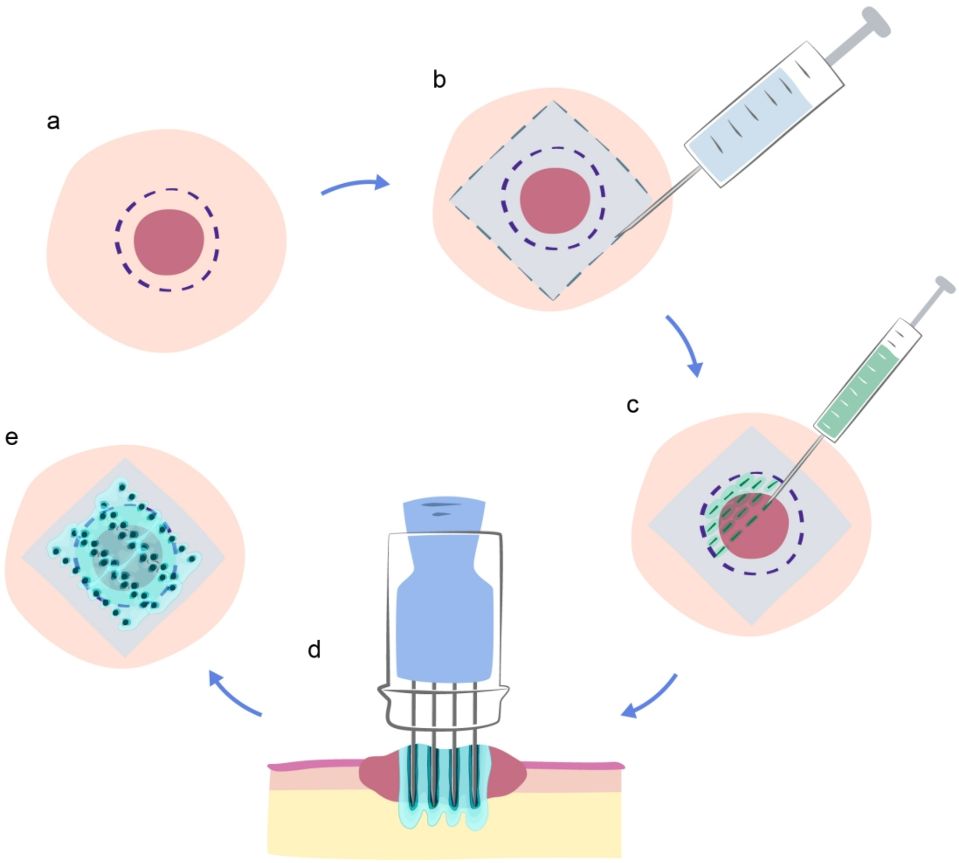

FIGURE 5Calcium electroporation procedure (a) The target area is defined as the area which is clinically visualized as tumour + a margin. (b) When performing the local anesthesia it is important to provide coverage of the margin as well as a zone around the margin so that the electrodes may be inserted without discomfort. Adding further local anesthetic below the tumour can also be helpful, in particular when treating larger lesions. (c) The calculated i.t. dose of calcium is injected into the tumour in a parallel fashion throughout the tumour. The margin area is then supplemented with calcium until the calcium is evenly distributed throughout the entire target area. (d) The electrode is inserted so that needles reach just beyond the deepest part of the tumour, and a pulse sequence is applied. The electrode can then be subsequently inserted in a systematic way to cover the entire tumour volume, as indicated in (e) As can be seen, the treatment area then covers the tumour with treatment margin.

170x157mm (300 x 300 DPI)

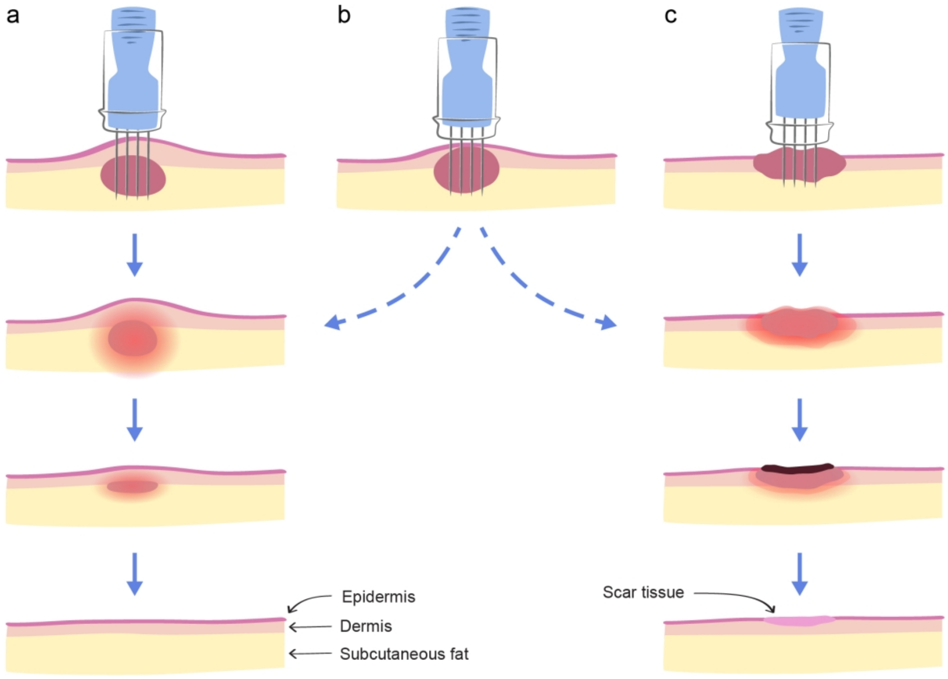

FIGURE 6
Cancer location in skin layers
Tumours may present as subcutaneous or cutaneous and involve some or all skin layers e.g. subcutaneous fat (a), dermis (b) and/or epidermis (c). If the epidermis is involved the tumours may present as ulcerating or fungating wounds.
Intact skin vs necrosis after CaEP. In most cases, subcutaneous tumours (a) will not ulcerate after calcium electroporation and skin will often appear intact after treatment. If treated tumours are situated as in (b) there may or may not be ulceration after treatment, depending on many factors such as patient healing potential and degree of invasion of the upper skin layers. Ulcerating or fungating tumours (c) may develop a necrotic, often crusted wound following treatment.

170x118mm (300 x 300 DPI)

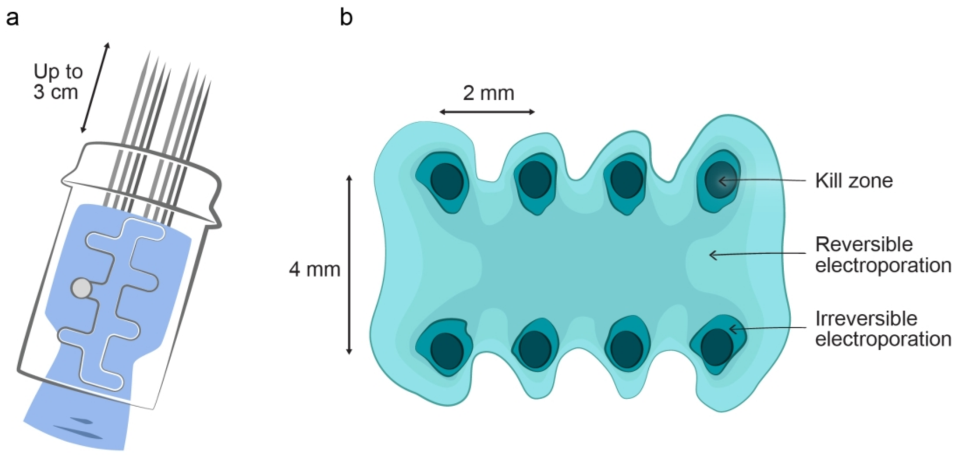

FIGURE 7Distribution of electric field(a)The linear array needle electrodes have needles of 0.7 mm that can be extended up to 3 cm. (b) A diagram of field distribution adapted from (Gehl et al, 46) shows that the distance between arrays is 4 mm, and distance between needles in the array app. 2 mm. At the needle insertion points, high fields will be present that may lead to irreversible electroporation. In the zone for reversible electroporation cell death will be due to effect of internalized calcium.

170x86mm (300 x 300 DPI)

SPIRIT

STANDARD PROTOCOL ITEMS: RECOMMENDATIONS FOR INTERVENTIONAL TRIALS

SPIRIT 2013 Checklist: Recommended items to address in a clinical trial protocol and related documents*

Section/item	Item No	Description	Addressed on page number
**Administrative information**			
Title	1	Descriptive title identifying the study design, population, interventions, and, if applicable, trial acronym	1
Trial registration	2a	Trial identifier and registry name. If not yet registered, name of intended registry	3
	2b	All items from the World Health Organization Trial Registration Data Set	NA
Protocol version	3	Date and version identifier	Clincaltrials.gov, see page 3
Funding	4	Sources and types of financial, material, and other support	18
Roles and responsibilities	5a	Names, affiliations, and roles of protocol contributors	1, 18
	5b	Name and contact information for the trial sponsor	1
	5c	Role of study sponsor and funders, if any, in study design; collection, management, analysis, and interpretation of data; writing of the report; and the decision to submit the report for publication, including whether they will have ultimate authority over any of these activities	18
	5d	Composition, roles, and responsibilities of the coordinating centre, steering committee, endpoint adjudication committee, data management team, and other individuals or groups overseeing the trial, if applicable (see Item 21a for data monitoring committee)	NA

For peer review only

**Introduction**			
Background and rationale	6a	Description of research question and justification for undertaking the trial, including summary of relevant studies (published and unpublished) examining benefits and harms for each intervention	6
	6b	Explanation for choice of comparators	NA
Objectives	7	Specific objectives or hypotheses	8
Trial design	8	Description of trial design including type of trial (eg, parallel group, crossover, factorial, single group), allocation ratio, and framework (eg, superiority, equivalence, noninferiority, exploratory)	7, 8
**Methods: Participants, interventions, and outcomes**			
Study setting	9	Description of study settings (eg, community clinic, academic hospital) and list of countries where data will be collected. Reference to where list of study sites can be obtained	7
Eligibility criteria	10	Inclusion and exclusion criteria for participants. If applicable, eligibility criteria for study centres and individuals who will perform the interventions (eg, surgeons, psychotherapists)	7
Interventions	11a	Interventions for each group with sufficient detail to allow replication, including how and when they will be administered	9-12
	11b	Criteria for discontinuing or modifying allocated interventions for a given trial participant (eg, drug dose change in response to harms, participant request, or improving/worsening disease)	13
	11c	Strategies to improve adherence to intervention protocols, and any procedures for monitoring adherence (eg, drug tablet return, laboratory tests)	9
	11d	Relevant concomitant care and interventions that are permitted or prohibited during the trial	9
Outcomes	12	Primary, secondary, and other outcomes, including the specific measurement variable (eg, systolic blood pressure), analysis metric (eg, change from baseline, final value, time to event), method of aggregation (eg, median, proportion), and time point for each outcome. Explanation of the clinical relevance of chosen efficacy and harm outcomes is strongly recommended	8
Participant timeline	13	Time schedule of enrolment, interventions (including any run-ins and washouts), assessments, and visits for participants. A schematic diagram is highly recommended (see Figure)	9, 12-14

For peer review only - http://bmjopen.bmj.com/site/about/guidelines.xhtml

Sample size	14	Estimated number of participants needed to achieve study objectives and how it was determined, including clinical and statistical assumptions supporting any sample size calculations	14
Recruitment	15	Strategies for achieving adequate participant enrolment to reach target sample size	Clinicaltrials.gov, see page 3

Methods: Assignment of interventions (for controlled trials)

Allocation:			
Sequence generation	16a	Method of generating the allocation sequence (eg, computer-generated random numbers), and list of any factors for stratification. To reduce predictability of a random sequence, details of any planned restriction (eg, blocking) should be provided in a separate document that is unavailable to those who enrol participants or assign interventions	NA
Allocation concealment mechanism	16b	Mechanism of implementing the allocation sequence (eg, central telephone; sequentially numbered, opaque, sealed envelopes), describing any steps to conceal the sequence until interventions are assigned	NA
Implementation	16c	Who will generate the allocation sequence, who will enrol participants, and who will assign participants to interventions	NA
Blinding (masking)	17a	Who will be blinded after assignment to interventions (eg, trial participants, care providers, outcome assessors, data analysts), and how	NA
	17b	If blinded, circumstances under which unblinding is permissible, and procedure for revealing a participant's allocated intervention during the trial	NA

Methods: Data collection, management, and analysis

Data collection methods	18a	Plans for assessment and collection of outcome, baseline, and other trial data, including any related processes to promote data quality (eg, duplicate measurements, training of assessors) and a description of study instruments (eg, questionnaires, laboratory tests) along with their reliability and validity, if known. Reference to where data collection forms can be found, if not in the protocol	9-14
	18b	Plans to promote participant retention and complete follow-up, including list of any outcome data to be collected for participants who discontinue or deviate from intervention protocols	NA

Data management	19	Plans for data entry, coding, security, and storage, including any related processes to promote data quality (eg, double data entry; range checks for data values). Reference to where details of data management procedures can be found, if not in the protocol	8
Statistical methods	20a	Statistical methods for analysing primary and secondary outcomes. Reference to where other details of the statistical analysis plan can be found, if not in the protocol	13
	20b	Methods for any additional analyses (eg, subgroup and adjusted analyses)	Clinicaltrials.gov, see page 3
	20c	Definition of analysis population relating to protocol non-adherence (eg, as randomised analysis), and any statistical methods to handle missing data (eg, multiple imputation)	NA
**Methods: Monitoring**			
Data monitoring	21a	Composition of data monitoring committee (DMC); summary of its role and reporting structure; statement of whether it is independent from the sponsor and competing interests; and reference to where further details about its charter can be found, if not in the protocol. Alternatively, an explanation of why a DMC is not needed	17-18
	21b	Description of any interim analyses and stopping guidelines, including who will have access to these interim results and make the final decision to terminate the trial	17-18
Harms	22	Plans for collecting, assessing, reporting, and managing solicited and spontaneously reported adverse events and other unintended effects of trial interventions or trial conduct	17-18
Auditing	23	Frequency and procedures for auditing trial conduct, if any, and whether the process will be independent from investigators and the sponsor	17
**Ethics and dissemination**			
Research ethics approval	24	Plans for seeking research ethics committee/institutional review board (REC/IRB) approval	17-18
Protocol amendments	25	Plans for communicating important protocol modifications (eg, changes to eligibility criteria, outcomes, analyses) to relevant parties (eg, investigators, REC/IRBs, trial participants, trial registries, journals, regulators)	NA

For peer review only - http://bmjopen.bmj.com/site/about/guidelines.xhtml

Consent or assent	26a	Who will obtain informed consent or assent from potential trial participants or authorised surrogates, and how (see Item 32)	9
	26b	Additional consent provisions for collection and use of participant data and biological specimens in ancillary studies, if applicable	Clinicaltrials.gov, see page 3
Confidentiality	27	How personal information about potential and enrolled participants will be collected, shared, and maintained in order to protect confidentiality before, during, and after the trial	9
Declaration of interests	28	Financial and other competing interests for principal investigators for the overall trial and each study site	18
Access to data	29	Statement of who will have access to the final trial dataset, and disclosure of contractual agreements that limit such access for investigators	NA
Ancillary and post-trial care	30	Provisions, if any, for ancillary and post-trial care, and for compensation to those who suffer harm from trial participation	NA
Dissemination policy	31a	Plans for investigators and sponsor to communicate trial results to participants, healthcare professionals, the public, and other relevant groups (eg, via publication, reporting in results databases, or other data sharing arrangements), including any publication restrictions	18
	31b	Authorship eligibility guidelines and any intended use of professional writers	Vancouver guidelines will be implemented. No professional writers will be used.
	31c	Plans, if any, for granting public access to the full protocol, participant-level dataset, and statistical code	NA
**Appendices**			
Informed consent materials	32	Model consent form and other related documentation given to participants and authorised surrogates	Supplementary

For peer review only

Biological specimens	33	Plans for collection, laboratory evaluation, and storage of biological specimens for genetic or molecular analysis in the current trial and for future use in ancillary studies, if applicable	13-14

*It is strongly recommended that this checklist be read in conjunction with the SPIRIT 2013 Explanation & Elaboration for important clarification on the items. Amendments to the protocol should be tracked and dated. The SPIRIT checklist is copyrighted by the SPIRIT Group under the Creative Commons "Attribution-NonCommercial-NoDerivs 3.0 Unported" license.

For peer review only
